# HMG-CoA Reductase Inhibitors Relieve Endoplasmic Reticulum Stress by Autophagy Inhibition in Rats With Permanent Brain Ischemia

**DOI:** 10.3389/fnins.2018.00405

**Published:** 2018-06-19

**Authors:** Tao Zhang, Dan Lu, Wanyong Yang, Changzheng Shi, Jiankun Zang, Lingling Shen, Hongcheng Mai, Anding Xu

**Affiliations:** ^1^Department of Cardiology, The First Affiliated Hospital of Jinan University, Guangzhou, China; ^2^Department of Neurology and Stroke Center, The First Affiliated Hospital of Jinan University, Guangzhou, China; ^3^Clinical Neuroscience Institute, Jinan University, Guangzhou, China; ^4^Department of Radiology, The First Affiliated Hospital of Jinan University, Guangzhou, China

**Keywords:** atorvastatin, 3-methyladenine, autophagy, endoplasmic reticulum stress, brain ischemia

## Abstract

Exploring and expanding the indications of common clinical drugs, such as statins, is important to improve the prognosis of patients with permanent cerebral infarction. It has been suggested that reversing the defects in cellular autophagy and ER stress with statin therapy may be a potential treatment option for reducing ischemic damage. Male Sprague-Dawley rats underwent permanent middle cerebral artery occlusion (PMCAO) by electrocoagulation surgery. Atorvastatin (ATV, 10 mg/kg/day) or vehicle was administered intraperitoneally. Rats were divided into the vehicle-treated (SHAM), ATV pretreatment for MCAO (AMCAO), and 3-methyladenine (3MA) combined with ATV pretreatment (3MAMCAO) groups. Magnetic resonance imaging, as well as immunohistochemical and Western blot assessments, were performed 24 h after MCAO. Each ATV-treated group demonstrated significant reductions in infarct volume compared with that in the vehicle-treated group at 24 h after MCAO, which was associated with autophagy reduction and ER stress attenuation in neurons and neovascularization. Next, Western blotting was used to detect the levels of the autophagy-related proteins LC3B and P62 and of ER stress pathway proteins. However, 3MA significantly partially inhibited the ER stress pathway via limiting the autophagic flux in the AMCAO group. In conclusion, our results imply that the neuroprotective function of ATV depends on autophagic activity to diminish ER stress-related cell apoptosis in rats with PMCAO and suggest that compounds that inhibit autophagic activity might reduce the neuroprotective effect of ATV after brain ischemia.

## Introduction

Stroke is a leading cause of death and disability worldwide according to reports by the World Heart Association, and ischemic stroke is the most common form, accounting for 87% of all strokes (Bin Abdulhak et al., 2014). Although great progress had been made in vascular recanalization, only 8.57 ∼ 11.90% of patients were treated in the therapy time window (≤3 ∼ 4.5 h) with intravenous (IV) recombinant tissue plasminogen activator (rt-PA) and 11.75% with endovascular treatment from 2010 to 2014 (Tekle et al., 2012; Zhang et al., 2016b,a). Achieving the proper balance of statin therapy will improve the prognosis of ischemic stroke patients. In recent years, atorvastatin (ATV), an important 3-hydroxy-3-methylglutaryl-coenzyme A (HMG-CoA) reductase inhibitor (statin), was suggested to increase the odds of better primary outcomes compared to treatment without statins after thrombolysis (Cappellari et al., 2013, 2015). Additionally, some animal studies have reported the amelioration of ischemic brain damage via the downregulation of 12/15-lipoxygenase (LOX), p38 mitogen activated protein kinase (p38 MAPK) and cytosolic phospholipase A2 (cPLA2) expression (Cui et al., 2010) or the expression of high mobility group box-1 protein (HMGB1) and HMGB1 receptors [receptor for advanced glycation end products (RAGE) and toll-like receptor 4 (TLR4)] (Wang et al., 2010). However, additional work is necessary to improve the benefit of ATV in ischemic stroke patients. It has been suggested that reversing the defects in cellular autophagy and endoplasmic reticulum (ER) stress (Tripathi et al., 2016) with statin therapy may be a potential treatment option for reducing ischemic damage.

The ER plays an essential role in the lipid and protein biogenesis necessary for cellular homeostasis, and autophagy is induced by nutrient deprivation under ER stress conditions to protect cells against death. ER and autophagy are noteworthy for the link between intracellular metabolism and ischemia. A previous study demonstrated that the autophagy-related proteins microtubule-associated proteins light chain 3 (LC3B) and sequestosome 1 (SQSTM1, p62) were significantly upregulated in penumbra neurons after focal cerebral ischemia (Rami and Kogel, 2008), whether ischemia-induced autophagy was beneficial or detrimental (Kubota et al., 2010). ATV activated autophagy after ischemic stroke, inhibited apoptosis, and promoted recovery of neurological function. However, ATV was suggested to prevent apoptosis and attenuate nerve cell injury by acting through the protein kinase-like endoplasmic reticulum kinase (PERK) phosphorylation of the eukaryotic initiation factor 2 (eIF2a)/caspase-3 pathway (Yang and Hu, 2015), but there was no detection of other key factors of the ER stress pathway. In a recent review, ER stress can induce autophagy processes mainly through the following signaling pathways: inositol-requiring enzyme 1 (IRE1-α)/phospho-c-Jun N-terminal kinases (JNKs)/X box-binding protein-1 (XBP1), PERK-eIF2α-activating transcription factor 4 (ATF4), and activating transcription factor 6 (ATF6) (Wang et al., 2018). Furthermore, whether ATV intervenes in the connection between autophagy and ER stress and how to improve the protective effect by combination with other treatments remain to be studied.

Therefore, in this present study, we focused on the protective effects of ATV pretreatment on neurons, endothelium, astrocytes and microglia after brain ischemia through autophagic activity and ER stress and proposed the interplay between ER stress and autophagy in this situation.

## Materials and Methods

### Animal Model

All animal procedures were conducted in strict accordance with the recommendations of the National Institutes of Health Guidelines (NIH Publications No. 8023, revised 1978) for the Care and Use of Laboratory Animals. The experimental protocols were approved by Competent Ethics Committees of Jinan University. A total of 40 male Sprague-Dawley rats (250–300 g) were purchased from the Animal Experiment Center of Southern Medical University, Guangzhou, China. Twenty rats were subjected to middle cerebral artery occlusion (MCAO) and 8 rats served as sham-operated (SHAM) controls. Permanent MCAO (PMCAO) was performed by electrocoagulation as previously described (Zhu et al., 2016). Briefly, the rats were anesthetized with chloral hydrate (0.3 mg/kg) by intraperitoneal injection. Under an operating microscope, the left MCA was exposed through a burr hole and occluded by electrocoagulation. The SHAM animals underwent the same surgical procedures except for the electrocoagulation of the middle cerebral artery. The body temperature was maintained at 37 ± 0.5°C during the surgery. At 3 days before MCAO, rats in the ATV group (AMCAO) and vehicle group (each group *N* = 8) were intraperitoneally injected with 10 mg/kg of ATV (Sigma-Aldrich, Fluka, St. Louis, MO, United States) and isopycnic vehicle, respectively, and then continuously injected once a day for 3 days. 3-Methyladenine (3MA; Sigma-Aldrich, Fluka, St. Louis, MO, United States; 5 mmol/L) at a dosage of 4 mL/kg/day (3MAMCAO group, *N* = 4) and isopycnic vehicle were given by tail vein injection (Zhao et al., 2016) (dissolved in 1 mL of saline) 1 h before the intraperitoneal injection of ATV and vehicle every time according to the previous studies to inhibit autophagy in the 3MAMCAO group (Guo et al., 2017). Only one animal was excluded because of model failure in the 3MAMCAO group. All animals survived for 24 h after MCAO before the following experiments.

### Magnetic Resonance Imaging (MRI)

The MRI was conducted by a Discovery 750 3.0 T scanner with an 8-channel wrist coil (GE Healthcare, Milwaukee, WI, United States) before heart perfusion, and the other 3 rats in each group were used for Western blotting. Five rats in each group were randomly anesthetized with 10% chloral hydrate and placed in a prone position before scanning. All experimental animals underwent coronal T1-weighted imaging, T2-weighted imaging (T2WI), diffusion-weighted imaging (DWI), and T2^∗^-weighted imaging, with scan parameters as follows. A coronal T2 sequence [effective echo time (TE): 81 ms, TR: 4600 ms; plane resolution: 0.25 mm × 0.27 mm; 17 slices, 2.0 mm in thickness] was used for T2-derived infarct measurement. DWI scans using single-shot echo planar imaging (EPI) pulse sequence were applied. Imaging parameters for DWI acquisition included field of view (FOV) = 80 mm × 50 mm, slice thickness/spacing = 2/0 mm, repetition time (TR)/TE = 3000/70 ms, matrix = 96 × 128, number of excitations (NEX) = 4, *b*-values = 800 s/mm^2^, total scanning time = 42 s. T2^∗^-weighted signal change was assessed by the parameters TE: 4, 8, 12, 17, 21, 25, 29, 33, 37, 42, 46, 50, 54, 58, 63, and 67 ms, TR: 126 ms, matrix 160 × 160, FOV 80 × 80 mm, and 17 contiguous slices of 2.0 mm in thickness.

### Nissl Staining

Animals were perfused with 0.9% saline followed by 4% paraformaldehyde and 0.1% glutaraldehyde in 0.1 M phosphate buffer (pH 7.4). Brains were harvested and postfixed overnight at 4°C. Coronal sections (10 μm) were cut using a vibratome and underwent Nissl staining according to previous methods. Briefly, brain sections (10 μm) were stained with Cresyl violet solution (Beyotime, Shanghai, China) at 50°C for 45 min. After dehydration in serially diluted ethanol and clearing in xylene, images of the Nissl-stained sections were captured using a microscope (Leica, Germany), and quantitative analysis was performed using ImageJ software (National Institutes of Health, United States).

### Immunofluorescence

Immunofluorescent staining was performed as previously described (Garbuzova-Davis et al., 2014). Similar to the Nissl staining, before immunofluorescence, the animals were perfused with 0.9% saline followed by 4% paraformaldehyde and 0.1% glutaraldehyde in 0.1 M phosphate buffer (pH 7.4). Brains were harvested and postfixed overnight at 4°C. Coronal sections (10 μm) were cut using a vibratome. The sections were incubated with primary antibodies [mouse anti-P62 monoclonal antibody, 1:250, catalog number (cat#) MABC32, Merck Millipore, Hong Kong, China; rabbit anti-LC3B polyclonal antibody, 1:200, cat# 2775, Cell Signaling Technology (CST), Danvers, MA, United States; rabbit anti-CD34 monoclonal antibody, as a type I transmembrane glycophosphoprotein expressed by vascular endothelium, 1:100, cat# ab8536, Abcam, Shanghai, China; mouse anti-glial fibrillary acidic protein (GFAP) monoclonal antibody, cat# ab4648, Abcam; mouse anti- ionized calcium-binding adapter molecule 1 (Iba-1), as a marker of microglia, monoclonal antibody, cat# sc32725, Santa Cruz, Shanghai, China] at 4°C overnight, washed with 0.01 PBS for 3 min × 5 min, and then incubated with secondary antibodies (488 nm FITC-labeled goat anti-rabbit IgG, 546 nm TRITC-labeled goat anti-mouse IgG, 1:1000 and 305 nm-labeled goat anti-mouse IgG; Yeasen, Shanghai, China) for 2 h at room temperature. After immunostaining, the sections were counterstained with 4′,6-diamidino-2-phenylindole (DAPI, 1:10000, Beyotime Biotechnology, Shanghai, China) to detect cell nuclei, and the slices were photographed under a fluorescence microscope (Leica DM1000, Germany).

### Transmission Electron Microscopy (TEM)

Fresh peri-infarct brain tissue samples (approximately 1 mm^3^) were fixed in 2.5% glutaraldehyde at 4°C overnight when the brain tissues were collected for Western blotting. Ultrathin sections were cut from the fixed blocks, dehydrated, embedded in epoxy resin, cut off, which is a standard process, and finally examined using an electron microscope (JEM-1200, JEOL Ltd., Tokyo, Japan) at 80 kV. Moreover, three areas in the ipsilateral peri-infarct cortex in each section were chosen.

### Western Blotting

Western blotting was performed as previously described (Lu et al., 2015). Fresh peri-infarct brain tissues were lysed with cell lysis buffer containing a protease inhibitor (Beyotime, Shanghai, China). The protein concentration was determined using a bicinchoninic acid protein assay kit (Beyotime, Shanghai, China). Samples containing equal amounts of protein (30 μg) were separated via sodium dodecyl sulfate-polyacrylamide gel electrophoresis and transferred to polyvinylidene difluoride membranes for blotting with antibodies purchased from CST, Danvers, MA, United States β-actin, 1:1000, cat# 4970; pPERK, 1:1000, cat# 3179; the phospho-eIF2α (peIF2α), 1:1000, cat# 5324; ATF4, which functions in the PERK and eIF2α ER stress responsive pathway, 1:1000, cat# 11815; ATF6, which is a transcription factor that activates target genes for the unfolded protein response (UPR) during ER stress, 1:1000, cat# 65880; the phosphorylation of the transcription factors of the nuclear factor κB (pNF-κB), 1:1000, cat# 3033; phospho-p38 MAPK (pP38), 1:1000, cat# 4511; pJNK, 1:1000, cat# 4668; C/EBP-homologous protein (CHOP), which participates in programmed cell death of ER-stressed cells by promoting protein synthesis and oxidative stress inside the ER, 1:1000, cat# 2895; Bcl-2, 1:1000, cat# 3498; calnexin, which is a chaperone characterized by assisting protein folding and quality control to ensure that only properly folded and assembled proteins proceed further along the secretory pathway, 1:1000, cat# 2679; Bip, 1:1000, cat# 3177; the ER-residing protein endoplasmic oxidoreductin-1 (Ero1-Lα), which is an ER membrane-associated *N*-glycoprotein that promotes oxidative protein folding, 1:1000, cat# 3264; the nuclear factor-like 2 (Nrf2), which maintains cellular homeostasis through the regulation of basal levels of antioxidant response genes, 1:1000, cat# 12721; cleaved-caspase 3, which is a critical executioner of apoptosis, 1:1000, cat# 9664; hypoxia-inducible factor 1 (HIF1-α), which plays a critical role in the cellular response to hypoxia, 1:1000, cat# ab51608 from CST; phospho-mammalian target of rapamycin (pmTOR), which has been shown to be activated by rapamycin to prevent ER stress and induce autophagy (Tripathi et al., 2016)). Additional antibodies, including IRE1-α, which is proposed to be a proximal sensor for the UPR that transmits the unfolded protein signal across the ER membrane; phospho-IRE1-α (1:1000; cat# ab48187); and vascular endothelial cadherin (VE-cadherin), which is localized at the intercellular junctions of endothelial cells where it is thought to play a role in the cohesion and organization of intercellular junctions (1:200, cat# WL02033), were purchased from Abcam (Wanleibio, Shenyang, China). The relevant horseradish peroxidase (HRP)-conjugated secondary antibodies (Yeasen, Shanghai, China) were incubated for 1 h. The SuperSignal substrate (Wanleibio, Changchun, China) was used to visualize the immunoreactive proteins. A Western Imaging System (Tanon 5200, Shanghai, China) and Quantity One software were used to measure the protein density. The density of protein bands was exposed and analyzed with a Tanon 2500 Gel Imaging System (Tanon, Shanghai, China). The raw data was submitted in the **Supplementary Data Sheet S1**.

### TTC (2, 3,5-Triphenyltetrazolium Chloride) Staining

Twenty-four hours after MCAO, 4 rats in each of the PMCAO, AMCAO, and 3MAMCAO groups were sacrificed. The brains were cut into 3-mm coronal slices, placed in 2% TTC (Sangon Biotech, Shanghai, China) solution, and incubated at 37°C for 5 min in the dark. Healthy tissues in the TTC solution were dyed red, and infarct tissues were white. The stained sections were captured by a camera, the images were analyzed using ImageJ software and then, the percentage of infarct volume was calculated (Zhang et al., 2011).

### Statistical Analysis

The data are presented as the mean ± standard error of the mean (SEM). One-way analysis of variance (ANOVA) was used to compare the average values between the different groups and was followed by an appropriate least significant difference (LSD) *post hoc* test (if homogeneity of variance was determined) or Tamhane’s T2 *post hoc* test (if homogeneity of variance was not determined) using the SPSS statistical software package (version 13.0; SPSS, Inc., Chicago, IL, United States). *p* < 0.05 was considered to indicate a statistically significant difference.

## Results

### The Changes in Infarct Volume and Cells Surrounding Infarct Areas

**FIGURE 1:**
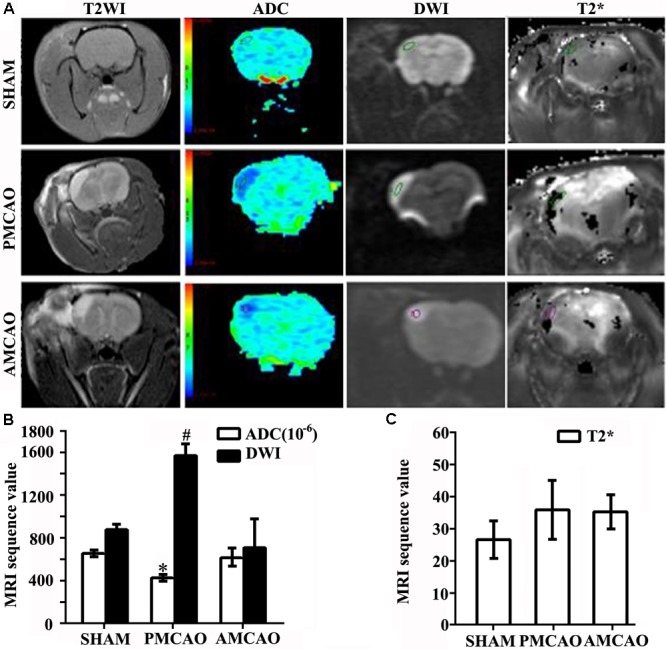
Infarct volumes. **(A)** MRI images showing changes in the ischemia area. Areas in pale blue in the ADC images represent the signal of the free diffusion of extracellular free water molecules; areas in dark blue reflect the limited diffusion of extracellular free water molecules. **(B)** Histogram showing changes in the values of the ADC and DWI in all groups. Quantitative analysis demonstrating the ADC values in the PMCAO group vs. the SHAM group (^∗^*p* < 0.05), the AMCAO group vs. the PMCAO group (^∗^*p* < 0.05), and the DWI values in the SHAM and AMCAO groups vs. the PMCAO group (^#^*p* < 0.01). Mean ± SEM, *N* = 5. **(C)** Histogram showing changes in the values of T2^∗^ in all groups. Mean ± SEM, *N* = 5.

Twenty-four hours after MCAO (*N* = 5), significant changes in signal intensity were observed in the ipsilateral hemisphere, as evaluated by DWI and the apparent diffusion coefficient (ADC). Compared to the matched area in the SHAM controls, the cerebral infarct area in the PMCAO animals revealed higher signals on T2-weighted imaging and on DWI and dark blue signals on the ADC maps, which were reversed to nearly normal signals by ATV pretreatment (**Figure 1A**). Quantitative analysis demonstrated that the DWI values were lower in the SHAM (878 ± 44.9) and AMCAO (790 ± 184) groups than in the PMCAO group (1575 ± 100.3, *p* < 0.01). The ADC value in the PMCAO group (426 ± 22.4) was lower than that in the SHAM group (653 ± 28.0, *p* < 0.05), and the ADC value was higher in the AMCAO group (621 ± 80.6) than in the PMCAO group (*p* < 0.05, **Figure 1B**). Moreover, there was no significant difference in the blood leakage levels obtained at 24 h post-MCAO by T2^∗^-weighted imaging among the three groups (**Figure 1C**).

**FIGURE 2:**
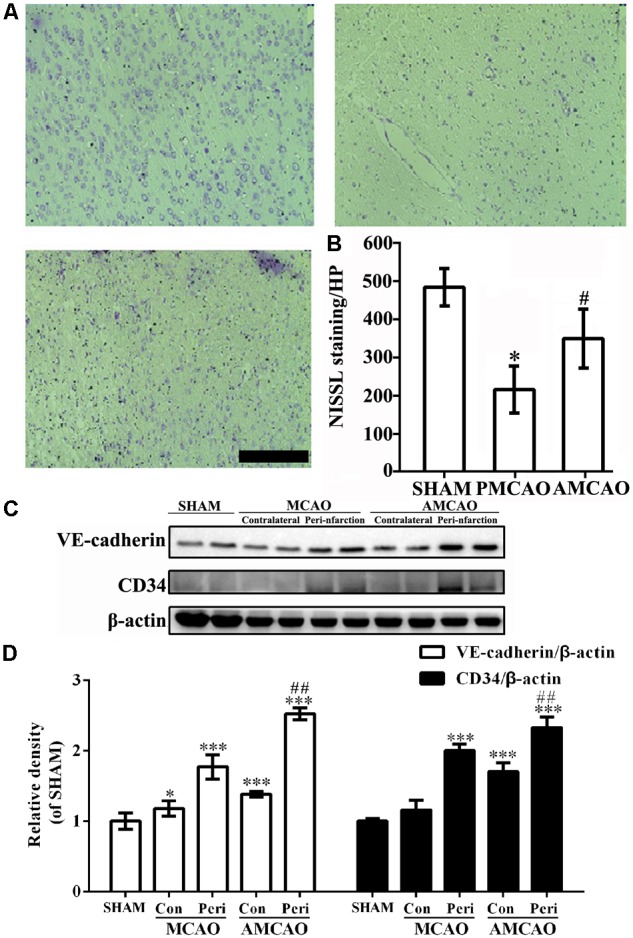
The changes in neurons and endothelial cells surrounding the infarct areas. **(A)** The number of surviving neurons shown by Nissl staining. Scale bar = 200 μm. **(B)** Quantitative analysis revealing the number of surviving neurons in the vehicle PMCAO group vs. the SHAM controls (^#^*p* < 0.01) and the AMCAO group vs. the vehicle PMCAO group (^∗^*p* < 0.05). Mean ± SEM, *N* = 5. **(C)** The protein bands of CD34, VE-cadherin and β-actin detected by Western blotting. SHAM indicates samples from the sham-operated group, contralateral indicates the samples from the contralateral tissue, and peri-infarction indicates the samples from the peri-infarct tissue of the infarct ipsilateral areas. **(D)** The relative density of CD34/β-actin and VE-cadherin/β-actin in the PMCAO group assessed in comparison with the SHAM group (^∗^*p* < 0.05, ^∗∗∗^*p* < 0.001) and the AMCAO group (Con, the contralateral tissue; Peri, the peri-infarct tissue of the infarct ipsilateral areas) vs. the PMCAO group (Peri), ^##^*p* < 0.01. Mean ± SEM was calculated by one-way analysis of variance (ANOVA) followed by the least significant difference (LSD) *post hoc* test (homogeneity of variance was determined), *N* = 3.

Next, the number of intact cells in the injured brain areas at 24 h after PMCAO injury was significantly reduced in the MCAO-injured animals treated with normal saline (252 ± 68.7/mm^2^) compared with that in the SHAM controls (564 ± 53.6/mm^2^), as detected by Nissl staining (*p* < 0.01), and showed shrunken cell bodies and condensed nuclei. However, the cell loss was significantly mitigated in the MCAO-injured animals treated with ATV (407 ± 86.1/mm^2^) compared with that in the vehicle PMCAO group (*p* < 0.05, **Figures 2A,B**).

Additionally, the expression of CD34 and VE-cadherin proteins in the penumbra core of the MCAO group was significantly increased compared with that in the SHAM control group (^∗∗∗^*p* < 0.001, *N* = 3), while pretreatment with ATV significantly increased the levels of the CD34 and VE-cadherin proteins in the AMCAO animals compared to the vehicle-treated PMCAO animals (^##^*p* < 0.01, *N* = 3, **Figures 2C,D**).

**FIGURE 3:**
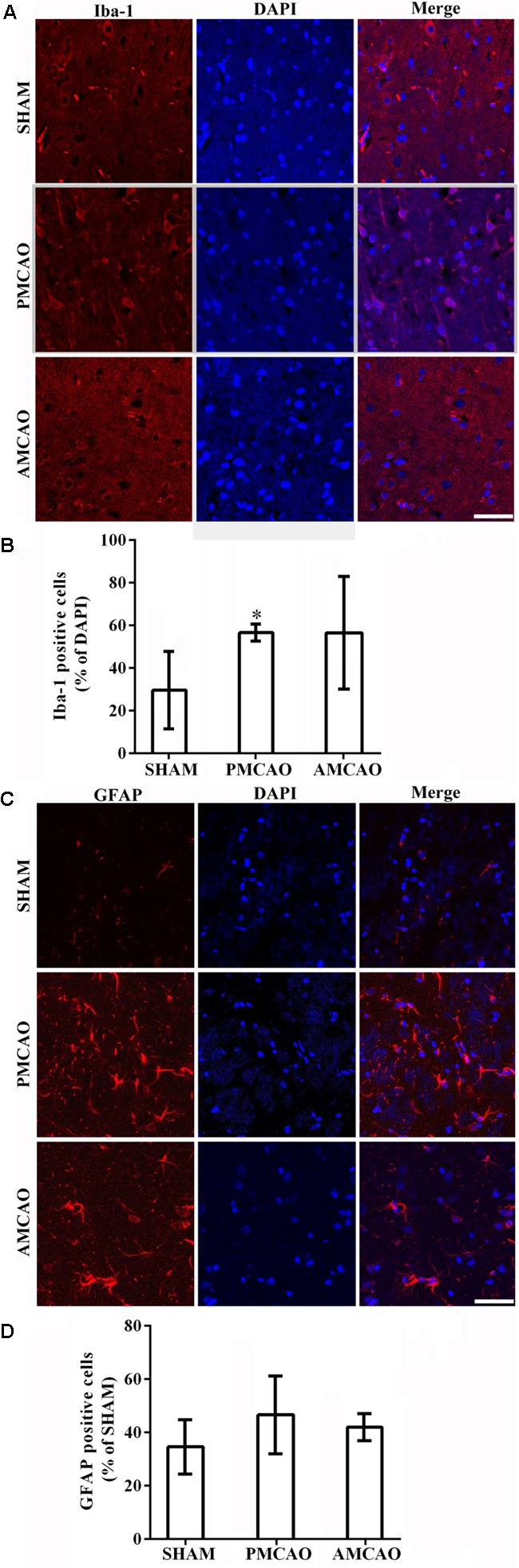
The changes in microglia and astrocytes surrounding the infarct areas. **(A)** Immunofluorescence micrographs showing differences in the number of Iba-1-positive cells (red) surrounding the infarct area in all experimental groups. Scale bar = 50 μm. **(B)** The Iba-1-labeled particle numbers (red)/DAPI (blue) in the PMCAO group vs. the SHAM group (^∗^*p* < 0.05). **(C)** Immunofluorescence micrographs showing differences in the number of GFAP-positive cells (red) surrounding the infarct area in all experimental groups. Scale bar = 50 μm. **(D)** The GFAP-labeled particle numbers (red)/DAPI (blue) detected in the SHAM, vehicle PMCAO, and AMCAO groups. Mean ± SEM, *N* = 5.

The quantitative analysis revealed that the percentage of the microglia marker Iba-1-positive cells (**Figure 3A**) was dramatically increased to 56.66 ± 3.97% in the peri-infarct area of the PMCAO-injured animals treated with normal saline compared with the percentage of 29.64 ± 18.21% in the SHAM control animals (*p* < 0.05, **Figure 3B**). There was no significant difference between the PMCAO-injured animals treated with ATV and the vehicle-treated PMCAO animals (**Figures 3A,B**). The analysis also revealed that the number of the astrocyte marker GFAP-positive cells (**Figure 3C**) was dramatically increased to 46.64 ± 14.60% in the peri-infarct area of the PMCAO-injured animals treated with normal saline compared with 34.62 ± 10.16% in the SHAM animals (*p* < 0.05, **Figure 3D**). There was no significant difference between the PMCAO-injured group treated with ATV and the vehicle PMCAO group (**Figures 3C,D**).

These findings were further confirmed in the animal experiment showing that pretreatment with ATV increased the expression of CD34 and VE-cadherin and the number of neurons in the PMCAO rats, and there was no significant difference in the number of glia cells between the PMCAO-injured group treated with ATV and the vehicle PMCAO group.

### Atorvastatin Promoted the Autophagic Activity of Vascular Endotheliocytes and Neurons

**FIGURE 4:**
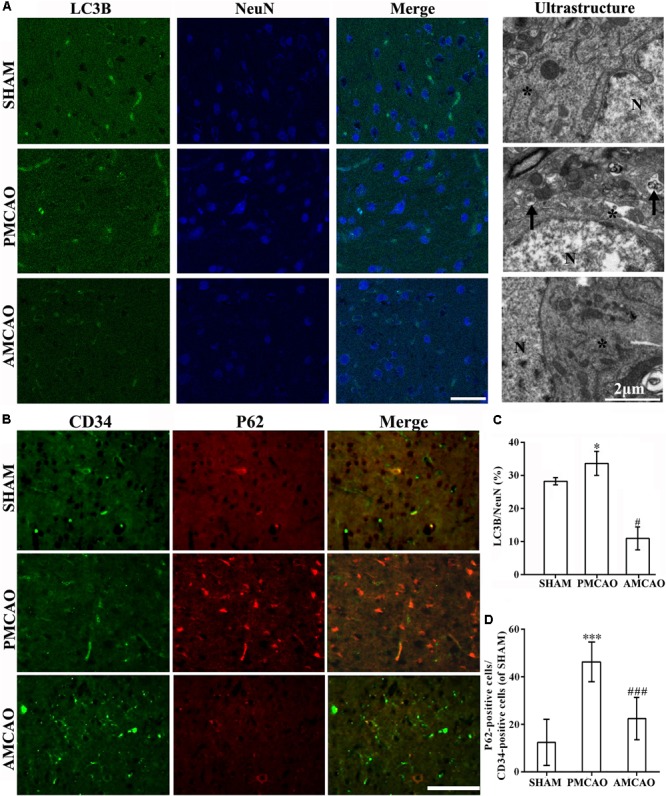
Protection of ATV pretreatment in vascular endothelial cells and neurons against ischemic brain injury by suppressing autophagic activity. **(A)** Immunofluorescence micrographs showing differences in the number of LC3B (red) and NeuN-positive cells (blue) in the ipsilateral injury area in all experimental groups. Scale bar = 50 μm. The ultrastructural results of each group shown by electron microscopy in the right column. The star, N and arrow, respectively, indicate the ribosomes and surrounding ribosomes, nucleus and autophagosomes. Scale bar = 2 μm. **(B)** Immunofluorescence micrographs showing the differences in the numbers of CD34- (green) and P62-positive cells (red) in the ipsilateral injury area in all experimental groups. Scale bar = 100 μm. **(C)** The LC3B-labeled particle numbers/NeuN detected in the PMCAO group vs. the SHAM group (^∗^*p* < 0.05) and the AMCAO group (^#^*p* < 0.05). Mean ± SEM, as calculated by one-way analysis of variance (ANOVA) followed by the least significant difference (LSD) *post hoc* test (homogeneity of variance was determined), *N* = 5. **(D)** The P62-labeled particle numbers/CD34-positive cells detected in the PMCAO group vs. the SHAM group (^∗∗∗^*p* < 0.001) and AMCAO group (^###^*p* < 0.001). Mean ± SEM was calculated by one-way ANOVA followed by LSD *post hoc* test (homogeneity of variance was determined), *N* = 5.

The numbers of LC3-positive cells (**Figure 4A**) and P62-positive endothelial cells (**Figure 4B**) in the brain sections were analyzed with the imaging software ImageJ. The number of LC3-positive cells in the peri-infarct areas in the ATV-pretreated group was also significantly less than that in the vehicle PMCAO group (^#^*p* < 0.05, **Figure 4C**). Then, TEM was used to examine the morphological changes in neurons. Cortical neurons from the SHAM control rats contained normal-looking nuclei with abundant ER and relatively healthy-looking mitochondria and lysosomes. In contrast, cortical neurons surrounding the infarct areas subjected to PMCAO injury displayed an increase in the number of autophagosomes, which were identified as bubble-like vacuoles enclosing recognizable cytoplasmic structures. In the AMCAO group, the appearance of neurons and their organelles were less damaged after pretreatment with ATV, and fewer autophagosomes were generated from the ER in the vehicle PMCAO group (**Figure 4A**, Ultrastructure). The endothelium cells were labeled with the CD34 antibody, and the results showed that the numbers of P62-positive endothelial cells in the SHAM controls (^∗∗∗^*p* < 0.001) and the peri-infarct areas of the ATV-pretreated groups (^###^*p* < 0.001) were significantly decreased compared with that in the vehicle PMCAO group (**Figures 4B,D**), suggesting that autophagy flux was blocked due to the failure of autophagosome degradation secondary to the P62 transition.

**FIGURE 5:**
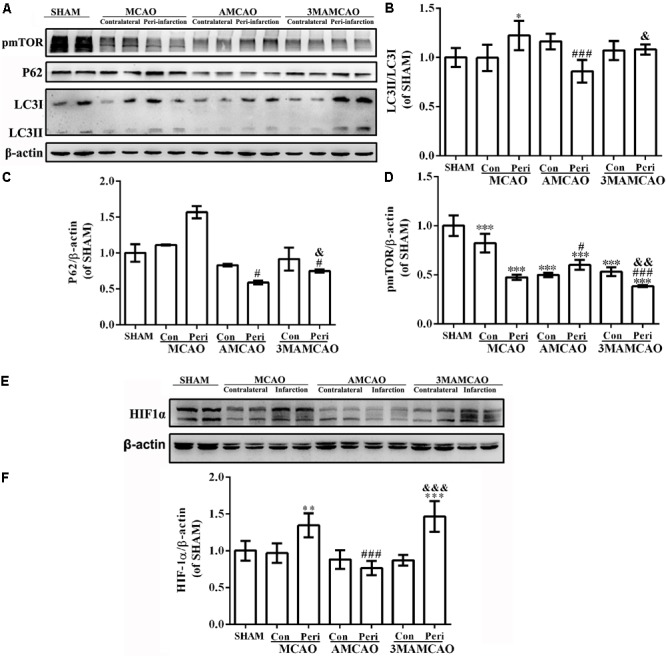
The protein levels of pmTOR, LC3B, P62, and HIF1α. **(A)** The protein bands of pmTOR, LC3B, P62, and β-actin detected using Western blotting and exposed by Tanon 2500/2500R. **(B)** The relative densities of LC3II/LC3I, **(C)** P62/β-actin, and **(D)** pmTOR/β-actin in the PMCAO group assessed in comparison with the SHAM group (^∗^*p* < 0.05, ^∗∗∗^*p* < 0.001), the AMCAO group vs. the PMCAO group (^#^*p* < 0.05, ^###^*p* < 0.001), and in the AMCAO group vs. the 3MAMCAO group (^&^*p* < 0.05, ^&&^*p* < 0.01). The mean ± SEM of LC3II/LC3I and pmTOR/β-actin was calculated by one-way analysis of variance (ANOVA) followed by the least significant difference (LSD) *post hoc* test (homogeneity of variance was determined), while the mean ± SEM of P62/β-actin was calculated by one-way ANOVA followed by Tamhane’s T2 test (homogeneity of variance was not determined), *N* = 3. **(E)** The protein bands of HIF1α and β-actin detected using Western blotting and exposed by Tanon 2500/2500R. **(F)** The relative density of HIF1α/β-actin in the PMCAO group assessed in comparison with the SHAM group (^∗∗^*p* < 0.01, ^∗∗∗^*p* < 0.01), the AMCAO group vs. the PMCAO group (^###^*p* < 0.001) and the AMCAO group vs. the 3MAMCAO group (^&&&^*p* < 0.001). The mean ± SEM of HIF1α/β-actin was calculated by one-way ANOVA followed by the LSD *post hoc* test (homogeneity of variance was determined), *N* = 3.

Activation of autophagy was further examined by Western blotting of LC3 and P62 proteins, two regulators in the autophagic cascade (**Figure 5A**). The results showed that the LC3II/LC3I ratio was increased in the vehicle PMCAO group (^∗^*p* < 0.05 vs. the SHAM controls, LSD test), and the trend of P62 was increased from the baseline level (Tamhane’s T2 test). The LC3II/LC3I conversion (**Figure 5B**, ^###^*p* < 0.001 vs. the vehicle PMCAO group) and the expression of P62 (**Figure 5C**, ^#^*p* < 0.05 vs. the vehicle PMCAO group) were further downregulated in the AMCAO group. In contrast, after treatment with the autophagy inhibitor 3MA, the ratio of LC3II/LC3I was decreased (**Figure 5B**, ^&^*p* < 0.05 vs. the AMCAO group) and the protein level of P62 was increased (**Figure 5C**, ^&^*p* < 0.05 vs. the AMCAO group). These results indicated that 3MA inhibited the autophagic flux (the increase of P62) in the 3MAMCAO group. In addition, pretreatment with ATV had a tendency to increase the pmTOR protein expression in the AMCAO group compared with that in the vehicle PMCAO group (^###^*p* < 0.001); however, 3MA reduced the phospho-mTOR expression in the 3MAMCAO group compared to that in the AMCAO group (^&&^*p* < 0.01) (**Figures 5A,D**). In the vehicle PMCAO group, HIF-1α, induced by hypoxia (Wang et al., 2017) and dependent on both mTORC1 and mTORC2 (Masoud and Li, 2015), was increased compared to those in the SHAM controls and AMCAO group (^∗∗^*p* < 0.01). In contrast, pretreatment with 3MA resulted in a significant increase of HIF-1α in the 3MAMCAO group compared to the level in the AMCAO group (^&&&^*p* < 0.001, **Figures 5E,F**). An explanation for this may be that ATV mainly relieves P62 expression in the vehicle PMCAO group to inhibit hypoxia damage, whereas 3MA ablated the effect of ATV on the P62 expression that would have affected the attenuation in autophagy.

### Autophagy Inhibition Affected ER Stress in Rats With ATV Pretreatment and PMCAO

**FIGURE 6:**
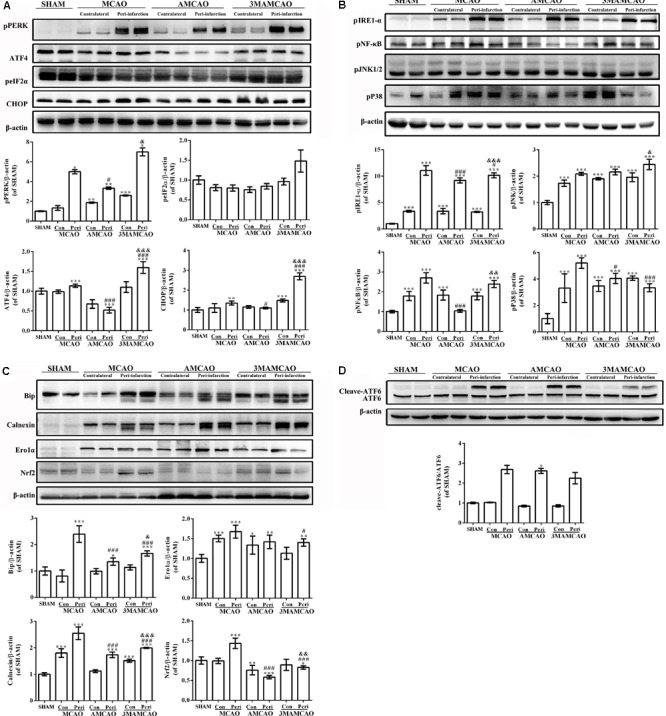
Regulation of protein expression in the ER stress pathway by ATV and 3MA in ischemic stroke rats. **(A)** The protein bands of pPERK, peIF2α, ATF4, CHOP, and β-actin detected by Western blotting and exposed by Tanon 2500/2500R. **(B)** The protein bands of ATF6 and β-actin detected by Western blotting and exposed by Tanon 2500/2500R. **(C)** The protein bands of pIRE-1α, pNF-κB, pP38, and pJNK detected using Western blotting and exposed by Tanon 2500/2500R. **(D)** The protein bands of Bip, calnexin, Ero1-Lα and Nrf2 detected using Western blotting and exposed by Tanon 2500/2500R. The relative densities of the other proteins were assessed by the ratio of each ER stress protein to β-actin in comparison with the SHAM group (^∗^*p* < 0.05, ^∗∗^*p* < 0.01, ^∗∗∗^*p* < 0.001), the AMCAO group vs. the PMCAO group (^#^*p* < 0.05, ^###^*p* < 0.001) and the AMCAO group vs. the 3MAMCAO group (^&^*p* < 0.05, ^&&^*p* < 0.01, ^&&&^*p* < 0.001). The mean ± SEM of pPERK/β-actin, peIF2α/β-actin, and cleaved-ATF6/ATF6 was calculated by one-way analysis of variance (ANOVA) followed by the Tamhane’s T2 test (homogeneity of variance was not determined), whereas the mean ± SEM of ATF4/β-actin, CHOP/β-actin, pIRE-1α/β-actin, pNF-κB/β-actin, pP38/β-actin, pJNK/β-actin, Bip/β-actin, calnexin/β-actin, Ero1-Lα/β-actin, and Nrf2/β-actin was calculated by one-way ANOVA followed by the least significant difference (LSD) *post hoc* test (homogeneity of variance was determined), *N* = 3.

Previous studies suggested that ischemia can activate ER stress (Drevinge et al., 2013). Herein, in the vehicle PMCAO group, the UPR was initiated, which is aimed at restoring the ER environment. In response to sustained ER stress, PERK activated ATF4 and phosphorylated eIF2α to increase the expression of CHOP. CHOP and eIF2α might provide an indicator of the cell condition and risk for apoptosis (Mantuano et al., 2011; Gharibani et al., 2013) (**Figure 6A**). In addition to PERK, the other two ER membrane-associated proteins that activate the UPR were ATF6 (**Figure 6D**) and IRE1, and the latter promotes the phosphorylation of JNK, P38, and NF-κB kinase (**Figure 6B**). However, pretreatment with ATV disturbed the activation of chaperones such as Grp78/Bip dissociation from IRE1-α, PERK, and ATF6 during acute ER stress (Yin et al., 2017), as well as calnexin, Nrf2 and Ero1-Lα, which participate in oxidative and Ca^2+^-related stress to regulate ER stress (Giorgi et al., 2015) (**Figure 6C**), and downregulated the downstream activation of JNK, P38 and NF-κB kinase (**Figure 6B**) to inhibit the subsequent apoptosis (Ma et al., 2017). Furthermore, there was no significance in the change in Ero1-Lα between the vehicle PMCAO and AMCAO groups (**Figure 6C**). Indeed, 3MA prevents autophagy at an early stage of autophagosome formation. Pretreatment with 3MA and ATV in the 3MAMCAO group altered the ER stress response compared to that in the AMCAO group. 3MA treatment in AMCAO rats increased the expression of PERK and the downstream protein IRE1-α, ATF4, CHOP, pNF-κB, and pJNK, as well as activated the chaperones Grp78/Bip, calnexin, and Nrf2. It was not clear whether 3MA treatment for AMCAO further decreased the activation of eIF2α, pP38, Ero1-Lα, or ATF6. In summary, ER stress could be partially upregulated after autophagy inhibition and partially downregulated via other proteins after 3MA application in the 3MAMCAO group. To determine the effect of the partially changed proteins, the key apoptosis and anti-apoptosis proteins were detected.

### Autophagy Inhibition Enhanced ER Stress-Related Apoptosis and Brain Injury

**FIGURE 7:**
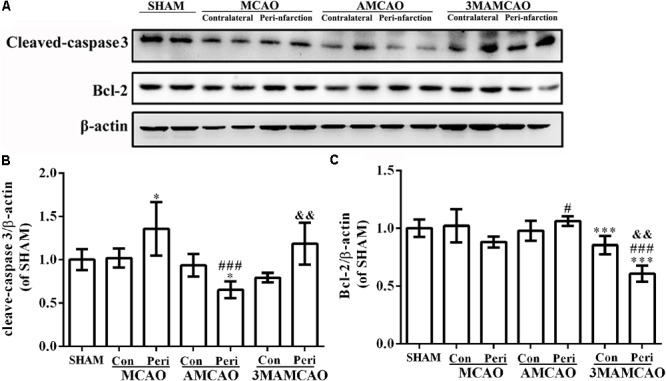
Regulation of protein levels of cleaved-caspase 3 and Bcl-2 by ATV and 3MA in ischemic stroke rats. **(A)** The protein bands of cleaved-caspase 3, Bcl-2 and β-actin detected by Western blotting. **(B)** The relative density of cleaved-caspase 3/β-actin in the PMCAO group assessed in comparison with the SHAM group (^∗^*p* < 0.05), and the AMCAO group vs. the PMCAO group (^###^*p* < 0.001) and the AMCAO group vs. the 3MAMCAO group (^&&^*p* < 0.01). The mean ± SEM of cleaved-caspase 3/β-actin was calculated by one-way analysis of variance (ANOVA) followed by the least significant difference (LSD) *post hoc* test (homogeneity of variance was determined), *N* = 3. **(C)** The relative density of Bcl-2/β-actin in the PMCAO group assessed in comparison with the SHAM group (^∗∗∗^*p* < 0.001) and the AMCAO group vs. the PMCAO group (^#^*p* < 0.05, ^###^*p* < 0.001) and the AMCAO group vs. the 3MAMCAO group (^&&^*p* < 0.01). The mean ± SEM of Bcl-2/β-actin was calculated by one-way ANOVA followed by the LSD *post hoc* test (homogeneity of variance was determined), *N* = 3.

**FIGURE 8:**
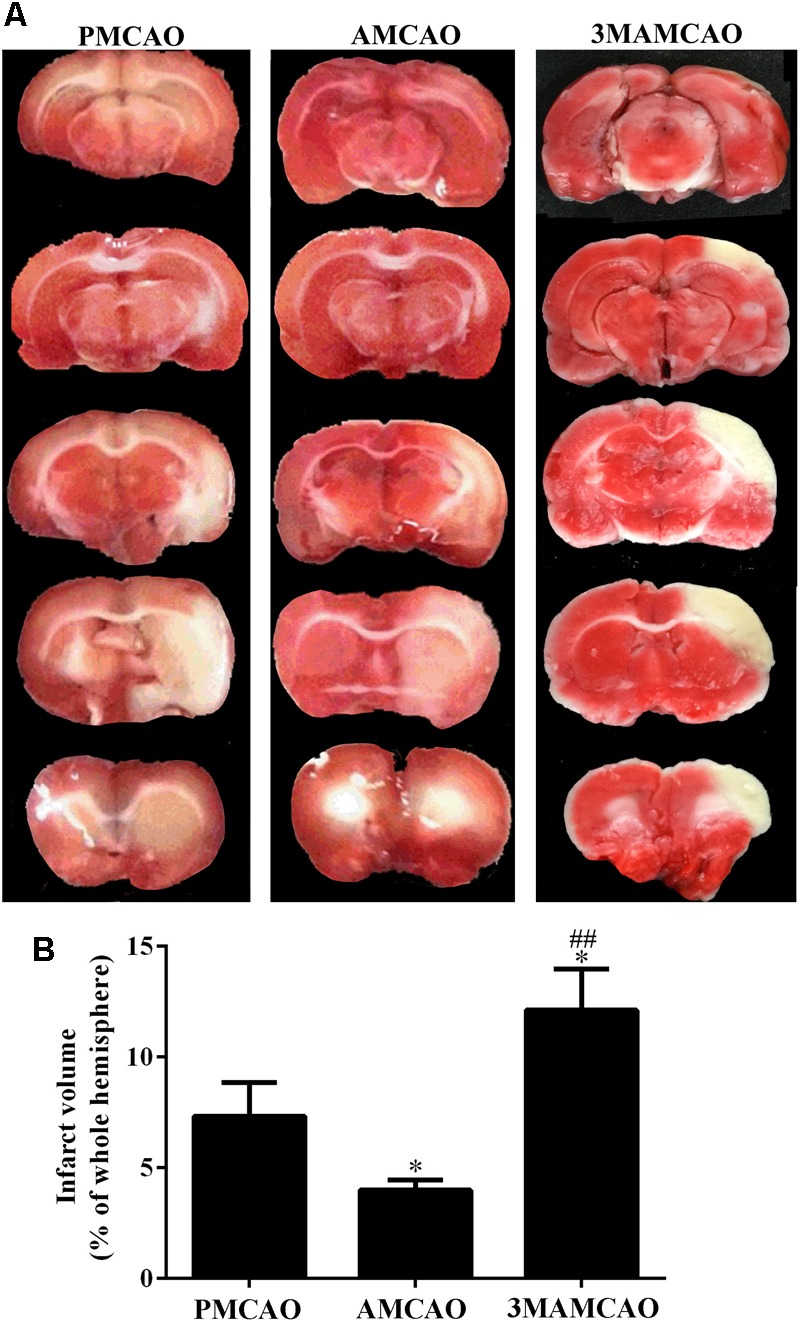
TTC staining. **(A)** Infarct volume ratios in the brains of the PMCAO, AMCAO and 3MAMCAO animals at 24 h after MCAO surgery. The white areas represent the infarct. **(B)** The percentages of infarct volume in the brains. *N* = 4, mean ± SEM ^∗^*p* < 0.05 vs. PMCAO. ^##^*p* < vs. AMCAO.

Having determined that ER stress activated caspase 3, we found that cleaved-caspase 3 was upregulated in the vehicle PMCAO group (^∗^*p* < 0.05 vs. the SHAM controls), but pretreatment with ATV significantly inhibited PMCAO-induced ER stress and the upregulation of cleaved-caspase 3 (^###^*p* < 0.001 vs. the PMCAO group). However, pretreatment of the autophagy inhibitor 3MA in the 3MAMCAO rats significantly promoted cleaved-caspase 3, indicating that autophagic inhibition hindered the effect of ATV pretreatment in ischemic rats (^&&^*p* < 0.01 vs. the AMCAO group, **Figures 7A,B**). On the other hand, pretreatment with ATV enhanced Bcl-2 in the vehicle PMCAO group (^#^*p* < 0.01 vs. the PMCAO group), while pretreatment with the autophagy inhibitor 3MA in the 3MAMCAO rats significantly reduced Bcl-2 expression (^&&^*p* < 0.01 vs. the AMCAO group, **Figures 7A,C**). Furthermore, the infarct volume ratio in the ATV-pretreated group was significantly decreased compared with that in the PMCAO group (^∗^*p* < 0.05). Furthermore, the infarct volume ratio in the 3MA-pretreated group was increased compared with that in the ATV-pretreated group (^##^*p* < 0.01) (**Figures 8A,B**). These findings suggested that the possible inhibition of autophagy resulted in a reduction in the protective effect of ATV pretreatment.

## Discussion

Guidelines for the prevention of stroke and transient ischemic attack recommend statin therapy, which should be initiated for the secondary prevention of patients to reduce the frequency of first and recurrent ischemic stroke. Although Hong et al. found that pretreatment with a low dose of ATV could protect neurons in the ischemic penumbra and reduce the infarct volume in MCAO-reperfusion rats, which depends on vascular recanalization, according to the survey results of the “Eleventh Five-Year Plan” key projects supported by National Science and Technology, the venous thrombolysis rate for cerebral infarction in China was only 1.3% (Tripathi et al., 2016) and approximately 10% abroad. Indeed, without urgent reperfusion management after MCAO, neurons ultimately advanced toward apoptotic death. Therefore, for most patients with cerebral infarction who are unable to receive vascular recanalization, whether or not statins can decrease the infarct size and increase the survival rate of normal brain cells is the focus of further research. The mechanisms underlying the protective effect of statins need to be further explored, which may expand the indications for statin treatment.

In this study, we investigated the damage changes in different cells in the peri-infarct areas. From MRI, we tentatively observed the infarct volume on DWI and ADC values and blood flow reflected by T2^∗^ in an MCAO rat brain (Liu et al., 2017). Vascular endotheliocytes were the first vulnerable cells compared with others following ischemia by toxic cytoplasmic calcium to affect the blood–brain barrier (BBB) integrity and neural cell viability (Posada-Duque et al., 2014). However, ATV treatment significantly limited the diffusion of water molecules and alleviated cytotoxic edema in this study. In addition, we found angiogenesis in the peri-infarct areas in the PMCAO rats, as well as increased numbers of astrocytes and microglia (Posada-Duque et al., 2014). In the MCAO rats that received ATV pretreatment, neurons and vascular endotheliocytes were promoted. In contrast, there was no significant difference in the number of microglia or astrocytes.

Under ultrastructural observation, damaged neurons were accompanied by autophagosomes and vacuolization of ER components. Recently, studies have reported that markedly increased autophagy following the upregulation of LC3 and downregulation of P62 in the peri-infarct areas following ischemia may reduce the infarct size, which was also validated in the vascular endotheliocytes and neurons in the AMCAO group. In comparison with the PMCAO control group, the number of vascular endothelial cells and neurons was higher after ATV pretreatment, and the ratio of P62-labeled cells/DAPI, P62 protein levels, the ratio of LC3-labeled neurons and LC3II/LC3I were decreased (Gao et al., 2012; Ma et al., 2017). Consistent with this phenomenon, 3MA pretreatment in the 3MAMCAO rats led to the stimulation of apoptosis and enlargement of the infarct, which were verified by the following results. LC3 as the main marker of autophagy flux provided a more accurate measurement of autophagic activity, and another marker, P62, specifically target ubiquitin-binding molecules for autophagic degradation and is inversely related to autophagic activity. When autophagy was initiated, LC3I was modified into the phosphatidylethanolamine-conjugated form LC3II, and LC3II was incorporated into autophagic vacuoles until degraded by lysosomes, whether P62 activation decided the downstream changes in autophagy (Yoshii and Mizushima, 2017). The autophagy processes in focal ischemia were disturbed by 3MA through the downregulation of LC3II and the upregulation of P62. Next, minimizing damage surrounding the ischemic areas requires the amelioration/prevention of cell apoptosis, which is the current vital therapeutic target in the treatment of acute ischemic stroke (Ma et al., 2017). In the current study, the hypoxia-related protein HIF1α and the apoptotic protein cleaved-caspase 3 were attenuated, and the anti-apoptotic protein Bcl-2 was activated by ATV compared to that in the PMCAO group. Our study demonstrated that interference with autophagy might inhibit the effect of ATV pretreatment. Thus, the neuroprotective function of ATV depends upon autophagic activity, and the combined therapy that inhibits autophagic activity would inhibit the neuroprotective effect of ATV after brain ischemia.

**FIGURE 9:**
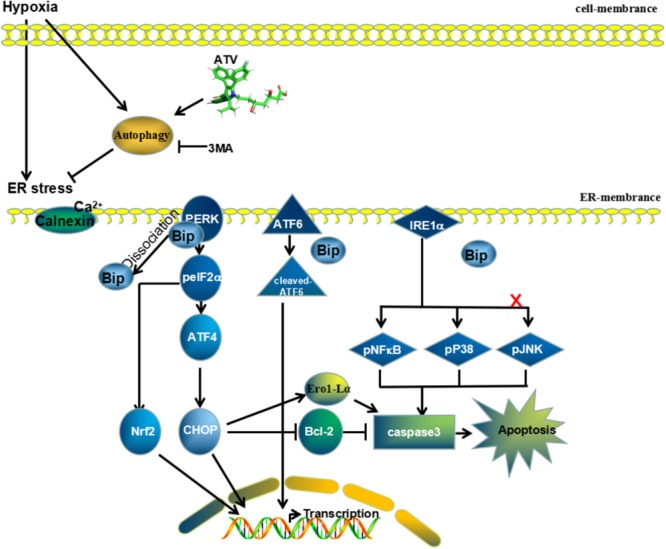
Mechanism of ATV effects on MCAO injury. Potential neuroprotective mechanisms of ATV pretreatment for permanent middle artery occlusion through downregulation of the ER stress-related pathways PERK/eIF2α/ATF4 and IRE-1/NF-κB by enhancing autophagy flux. Compounds such as 3MA that inhibit autophagic activity will reduce the neuroprotective effect of ATV after brain ischemia.

To further discuss autophagy-induced apoptosis, we found in the PMCAO groups that autophagosomes were triggered by ER swelling in the ischemic area, and in turn, autophagy also affects ER stress. Cross talking with UPR/ER effector molecules, LC3 canonically triggers the degradation of organelles/molecules in the cytosol and potentiates the adaptive ability of the cell by clearing the load of misfolded proteins from the ER lumen (Rahman et al., 2017) to affect apoptosis (Talma et al., 2016). ER stress is initiated through the IRE-1, PERK, and ATF6 pathways in mammalian cells (Nakka et al., 2016; Rahman et al., 2017). IRE1α from the ER might be involved in integrating ERS signaling with inflammatory-response signaling for activating both JNK and NF-κB (Wu et al., 2014) to trigger cell apoptosis. In some ways, the permanent brain ischemia significantly enhanced the ER stress through PERK/eIF2α/ATF4, ATF6 and IRE-1/NF-κB/JNK/p38 signaling. With ATV pretreatment, the molecular chaperone proteins were diminished, such as CHOP regulated by the mTOR pathway (Ma et al., 2018), Grp78 (Louessard et al., 2017), the calcium overloaded-related protein calnexin (Zhang et al., 2016b,a), and the oxidative system proteins Ero1-Lα (Capriotti and Murphy, 2016) and Nrf2 (Bocci and Valacchi, 2015; Zhang et al., 2017). However, the expression of pJNK and ATF6 was not significantly different between the AMCAO group and the vehicle PMCAO group. Cell apoptosis triggered by these proteins via MCAO stress could be inhibited by ATV pretreatment. The autophagy inhibitor, 3MA, could attenuate ATP generation, affect mTORC1 activation and lead to cell death in response to nutrition deprivation. It reduced the autophagy flux mainly via P62 upregulation. We supposed that autophagy might be the regulator of ER stress in statin-pretreated MCAO brains (**Figure 9**).

However, our study had several limitations. We only showed that the autophagy inhibitor 3MA potentially alters the neuroprotective effects of ATV *in vivo*; there was a lack of experiments to validate whether the autophagy activators benefit the protective effect of ATV. Future studies are needed to define the mechanism of autophagy activators for ATV therapy and the changes in glia in more animals.

These findings firmly indicated that the protective effects of ATV pretreatment were related to the reduction in autophagic activity and ER stress in vascular endothelial cells and neurons. In fact, the promotion of P62 significantly weakened the reduction in autophagic activity to diminish the ER stress in ATV pretreatment; in other words, our study provides a new view for ATV therapy, namely, that avoiding P62 hoarding or increasing autophagosome degradation might enhance the effect and expand the clinical indications of ATV in the treatment of acute ischemic stroke.

## Author Contributions

TZ completed the most draft writing. DL completed the most experiments. WY revised the draft. CS analyzed the MRI parts. JZ repeated the WB results. LS repeated the IF results. HM feed the rats. AX designed the whole experiments and draft, and submitted the manuscript to the Journal of Cellular Neuroscience.

## Conflict of Interest Statement

The authors declare that the research was conducted in the absence of any commercial or financial relationships that could be construed as a potential conflict of interest.

## Supplementary Material

DATA SHEET S1 The whole raw bands of Western Blot exposed using Tanon 2500 Gel Imaging System, including the bands of CD34, VE-cadherin in **Figure 2**, pmTOR, LC3B, P62, and HIF1α in **Figure 5**, ER stress pathway protein in **Figure 6**, and cleaved-caspase 3 and Bcl-2 in **Figure 7**.
