# Supplementary material for: HMG-CoA Reductase Inhibitors Relieve Endoplasmic Reticulum Stress by Autophagy Inhibition in Rats With Permanent Brain Ischemia
Source: Front Neurosci. 2018 Jun 19;12:405. doi: 10.3389/fnins.2018.00405 (PMC6018104; doi:10.3389/fnins.2018.00405)

Suplementary Figure (Original Figures)

VE-cadherin

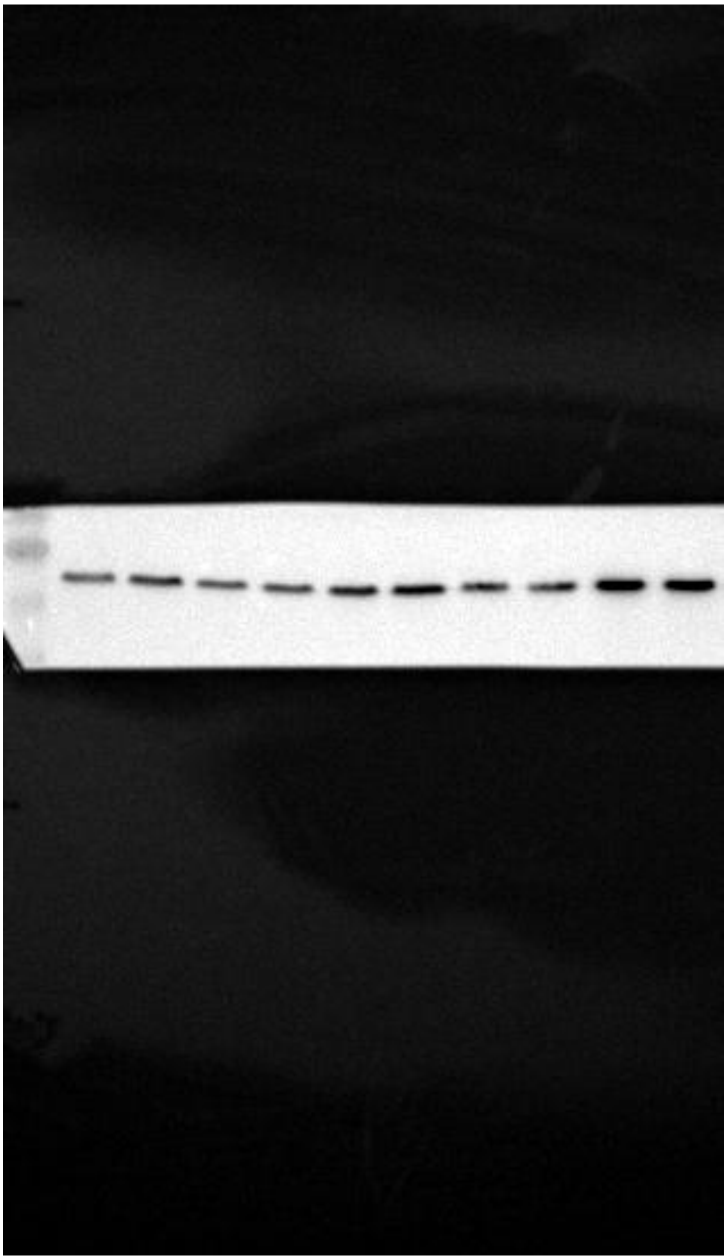

CD34

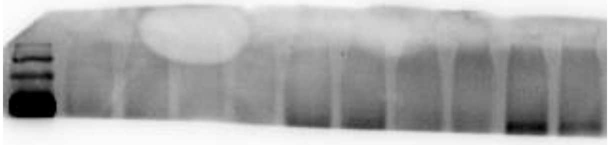

pmTOR

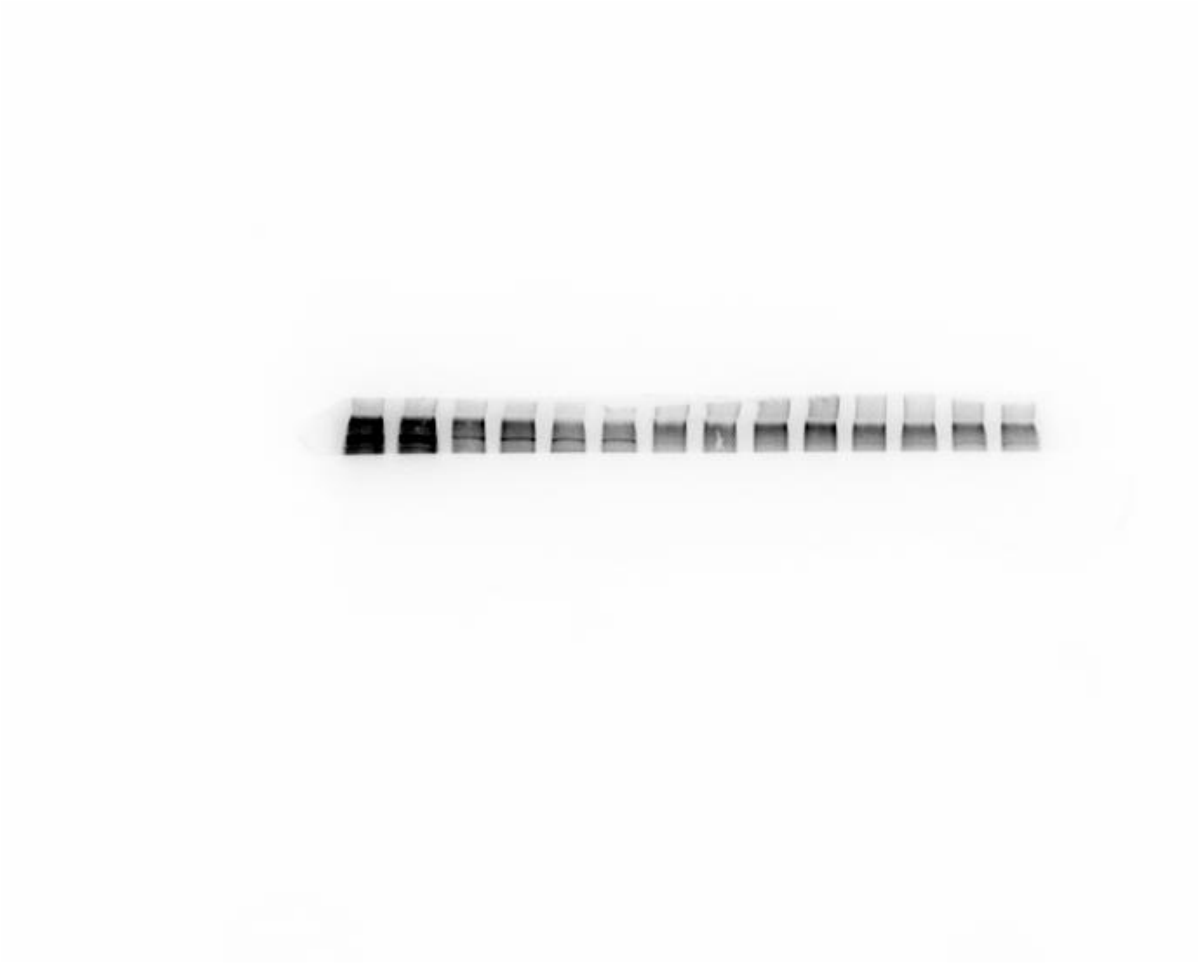

β-actin

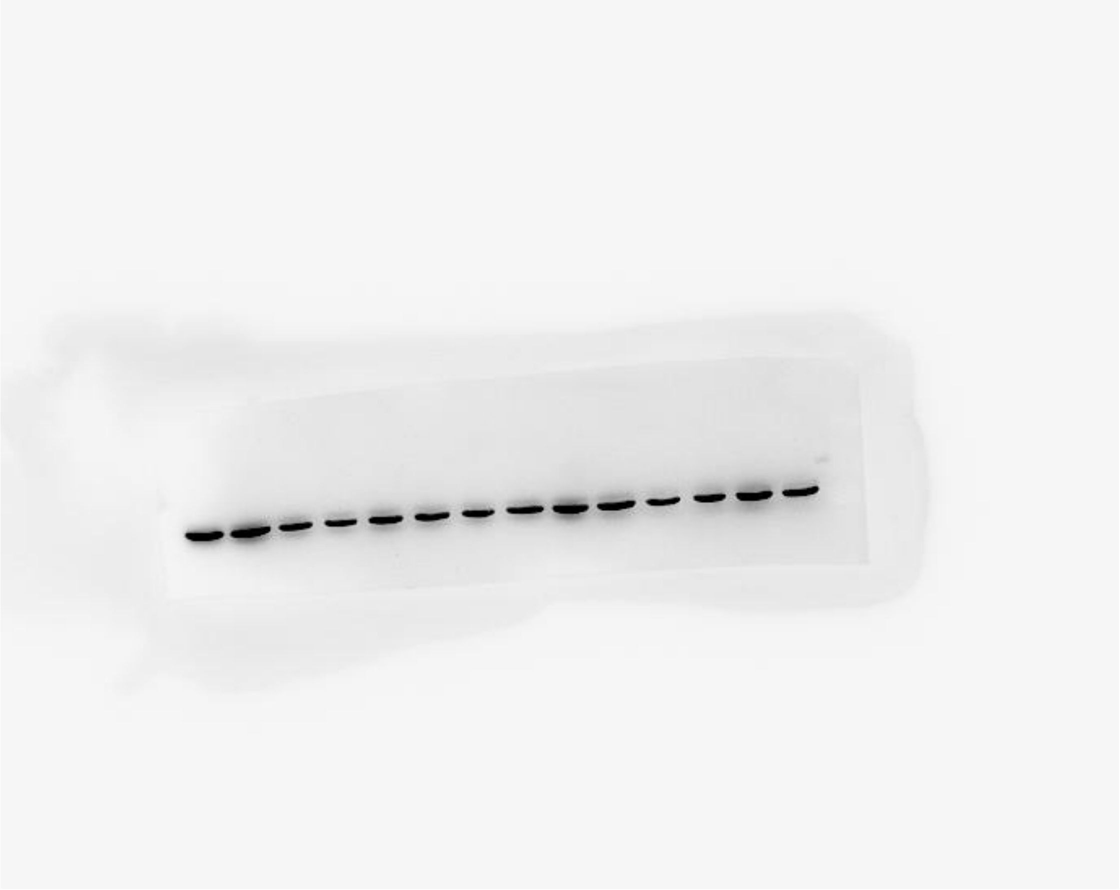

p62

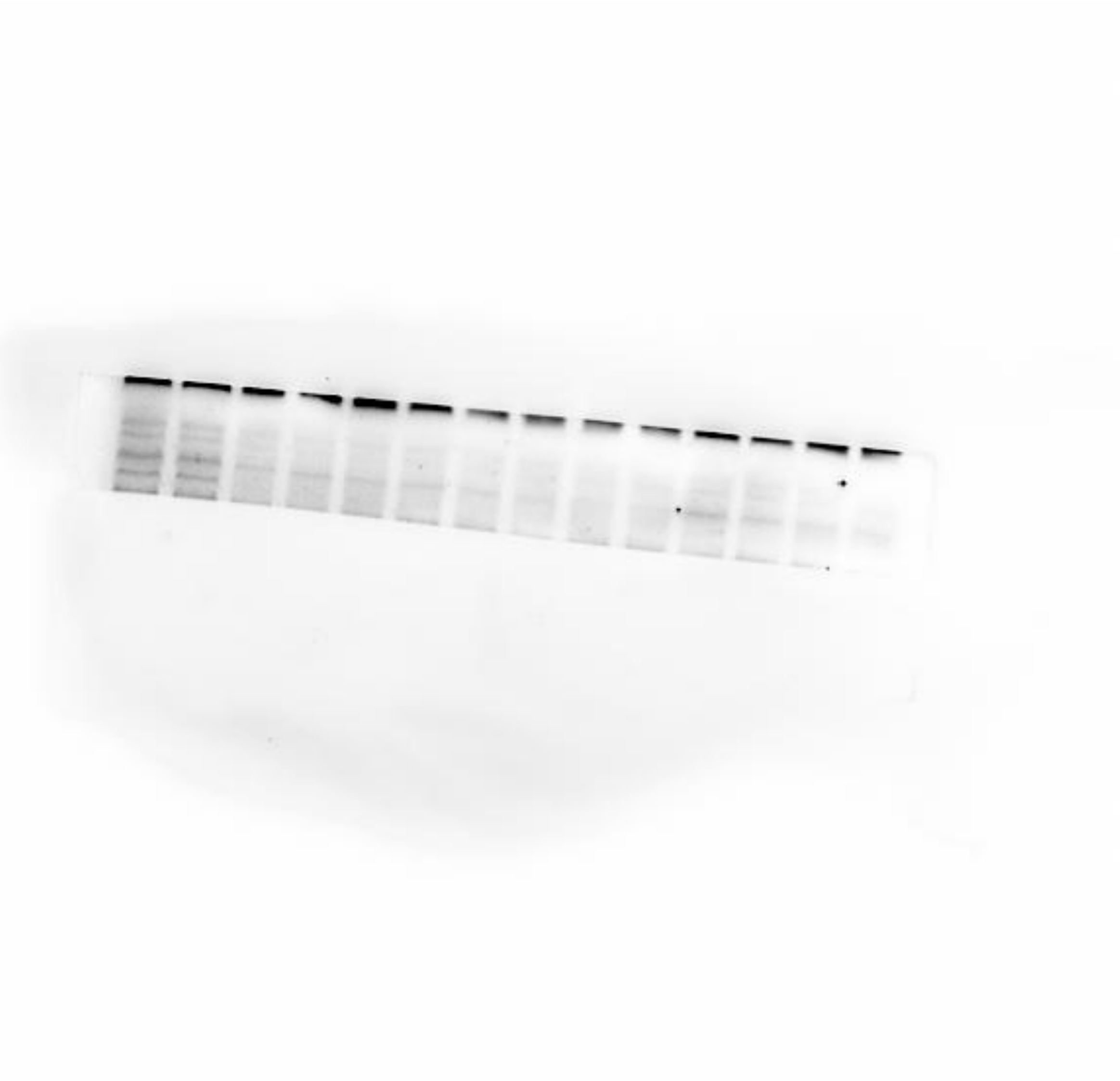

LC3B

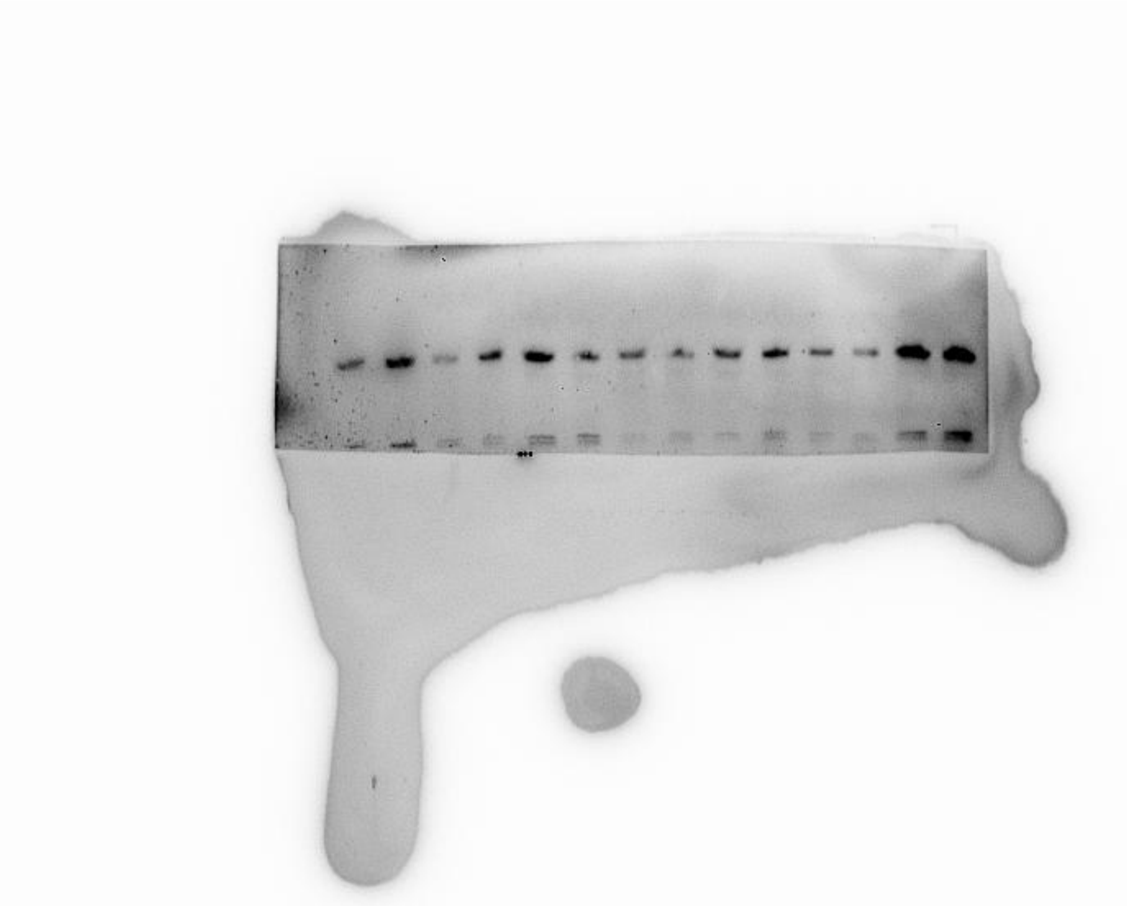

HIF1a

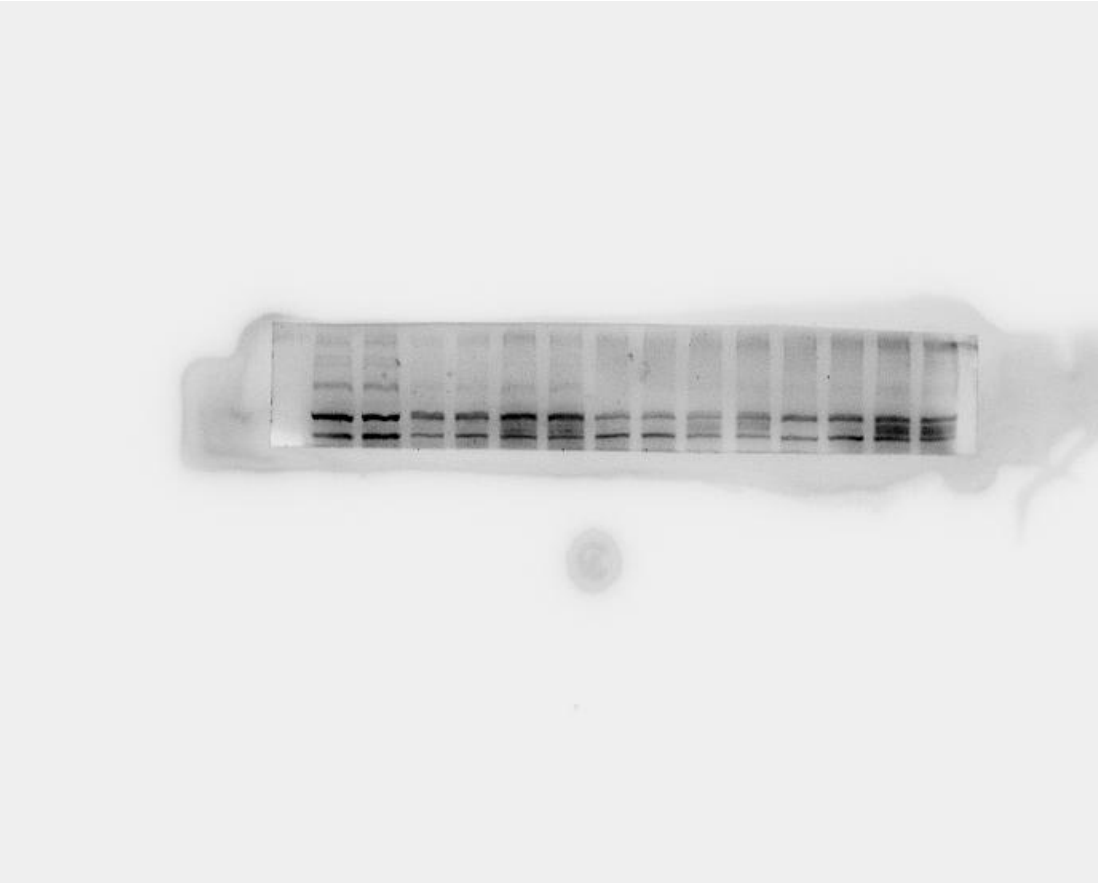

β-actin (Upper for Figure 5E, below for Figure 6A)

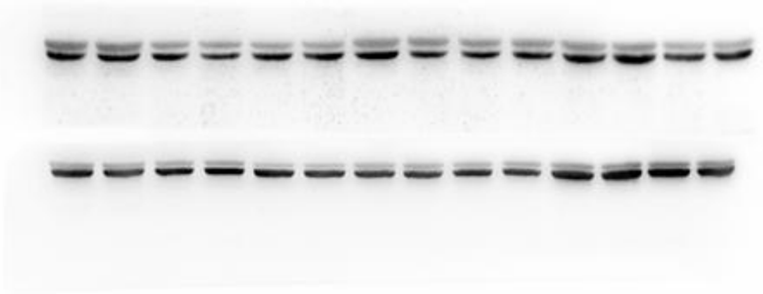

p-PERK

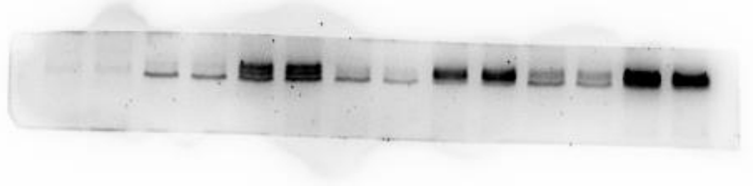

ATF4

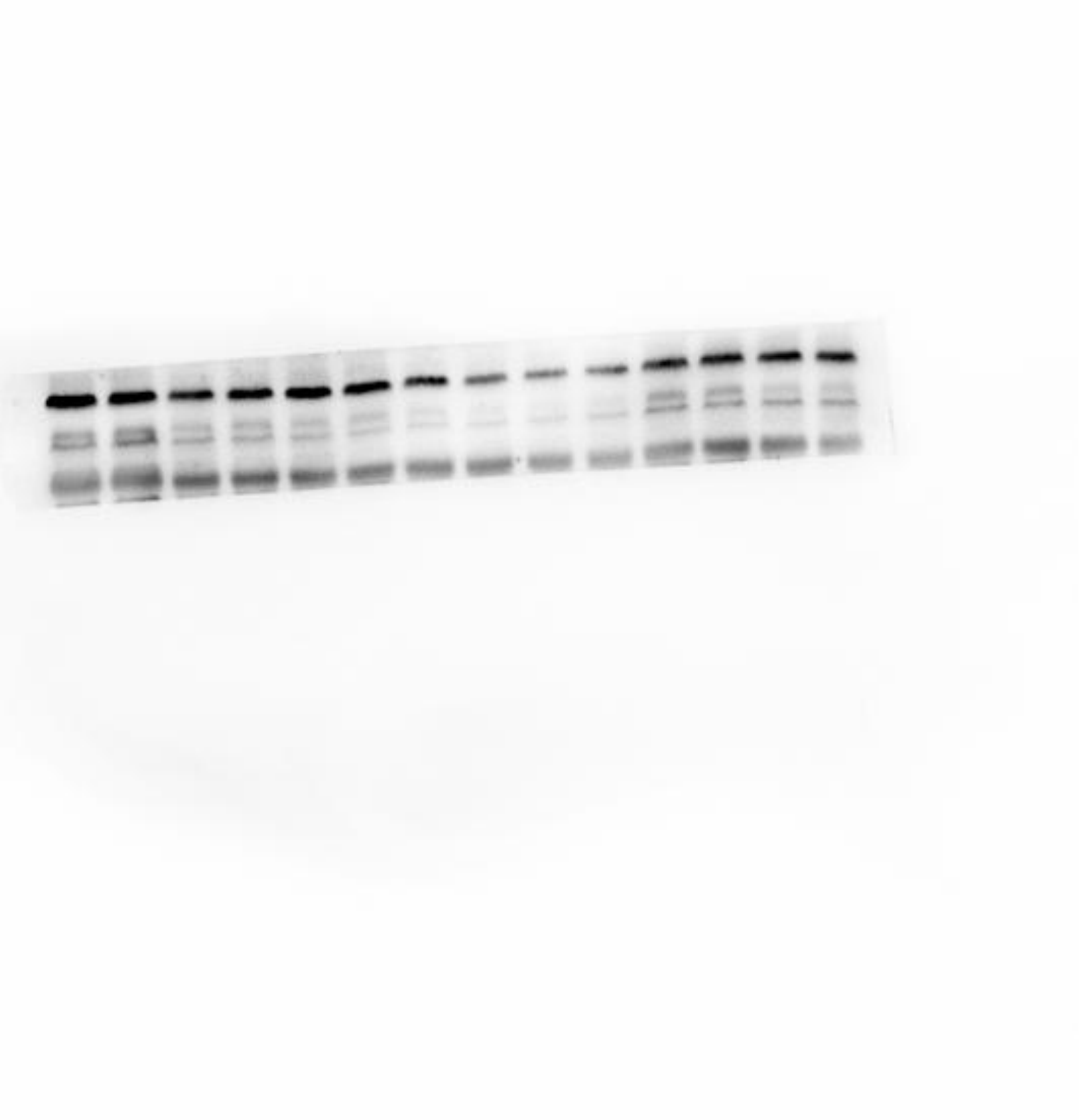

peIF2a

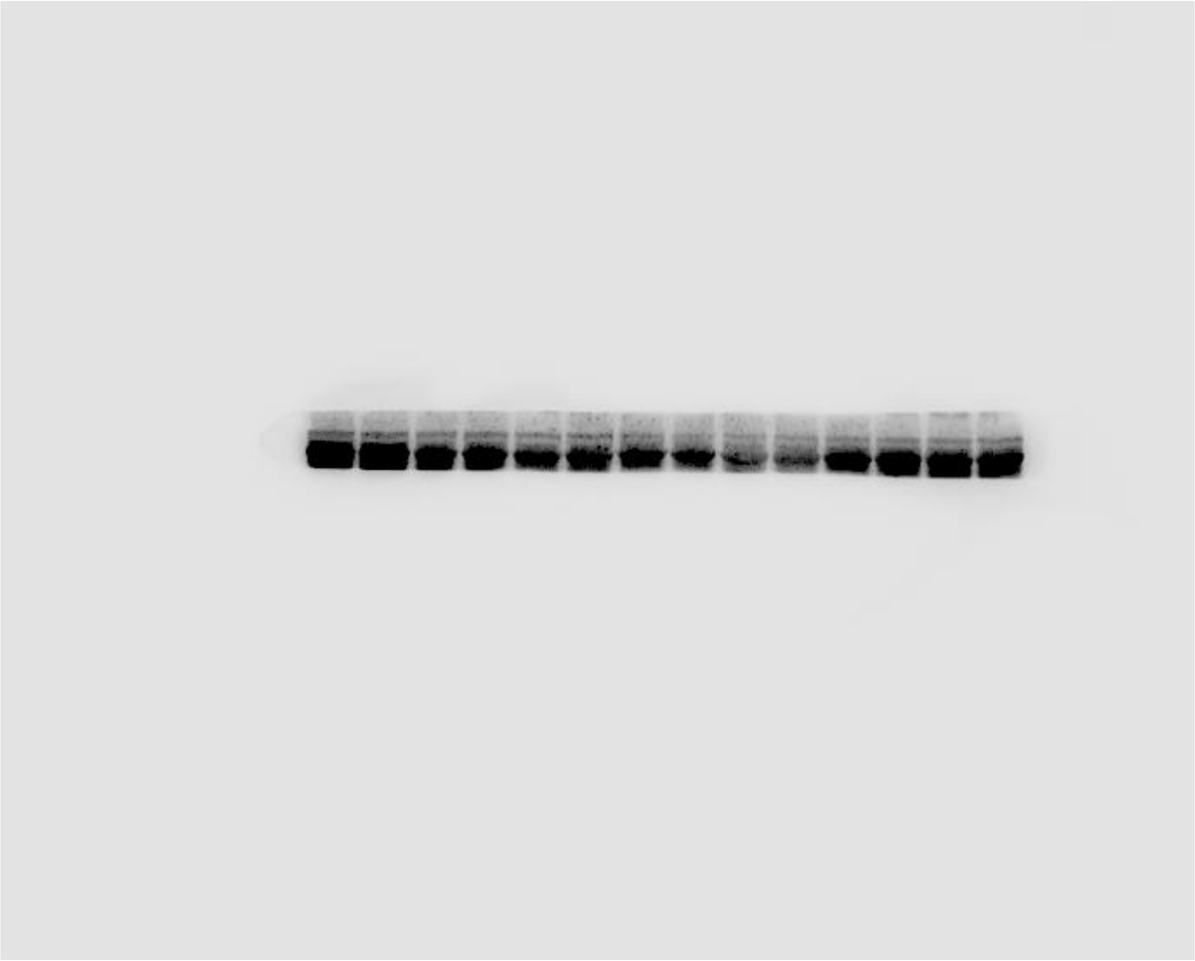

CHOP

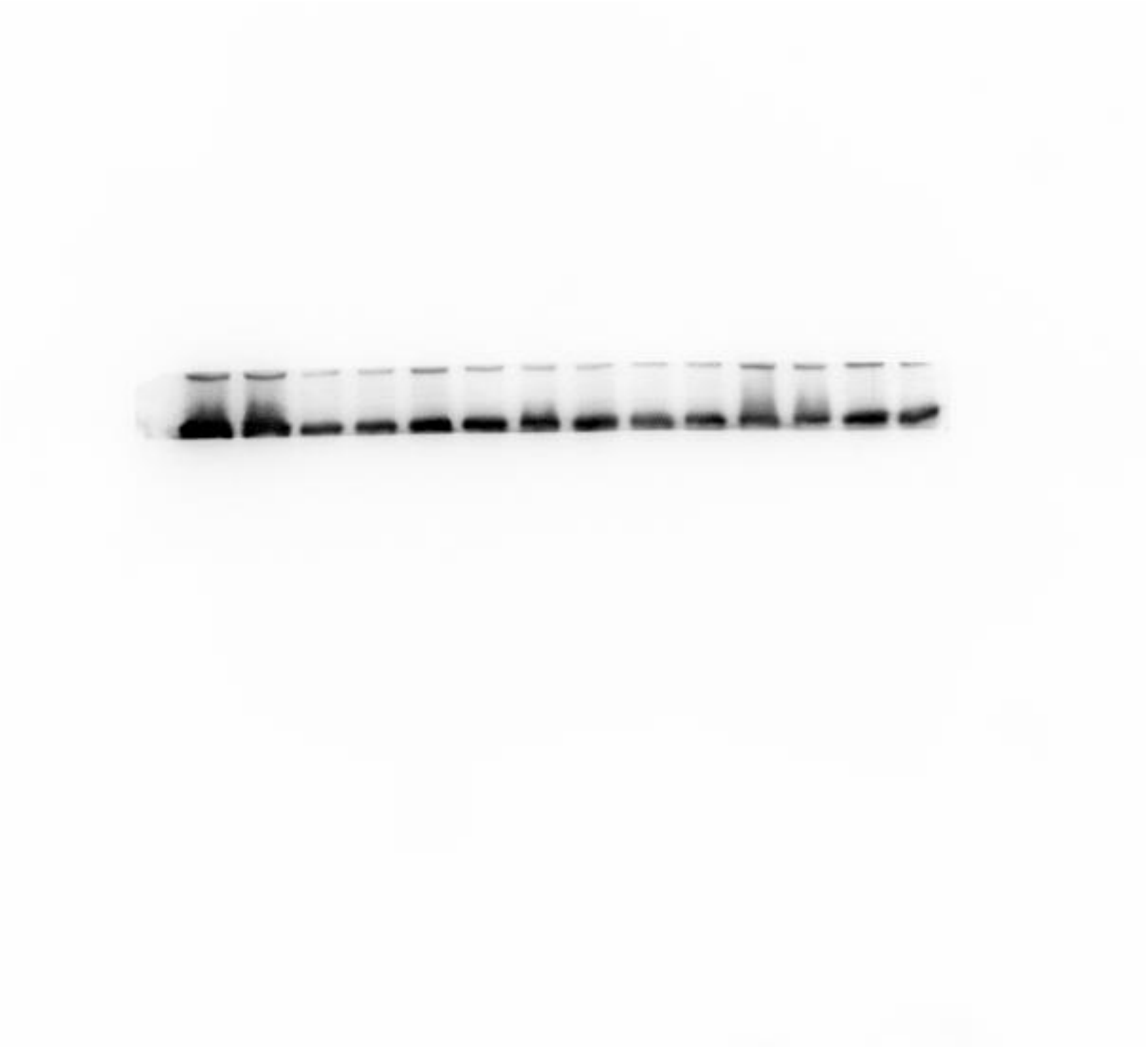

IRE1-a

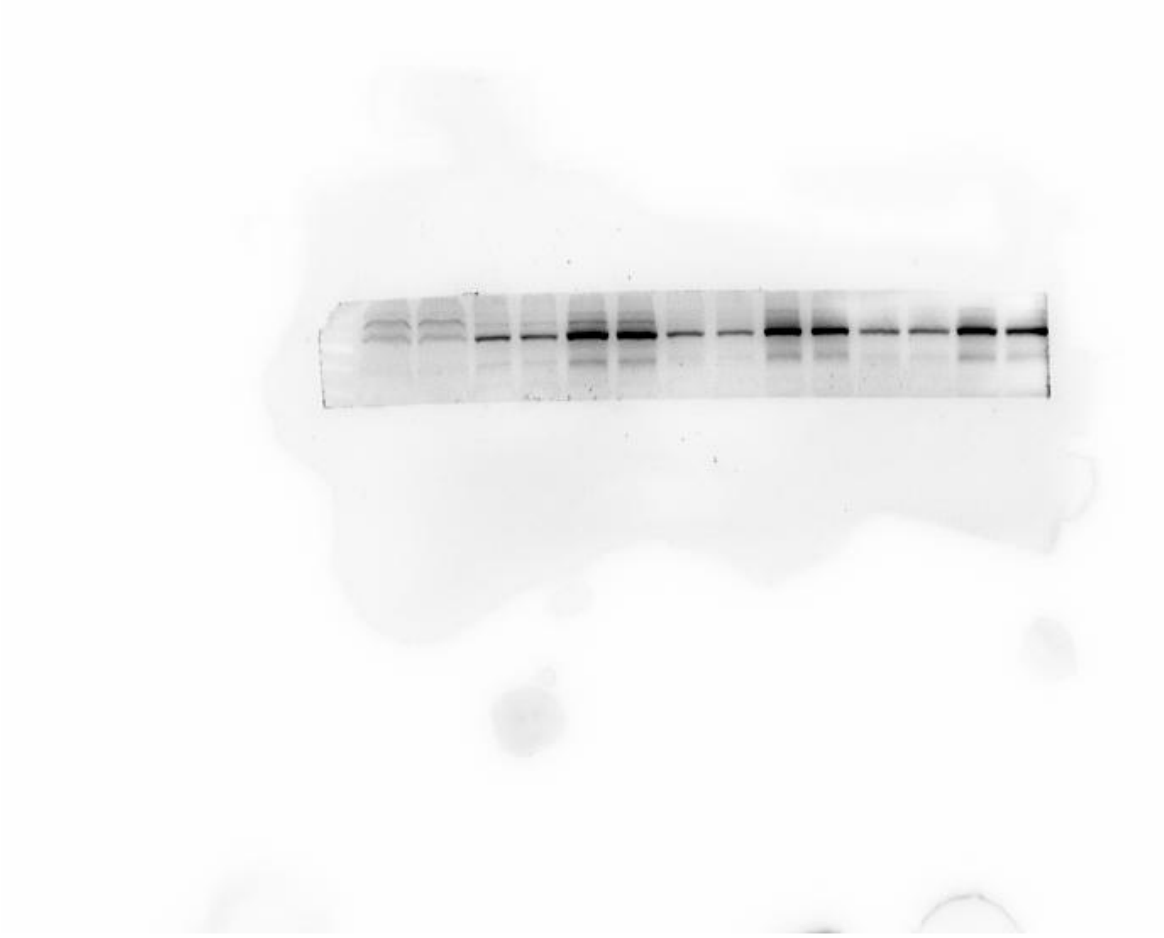

pNF-KB

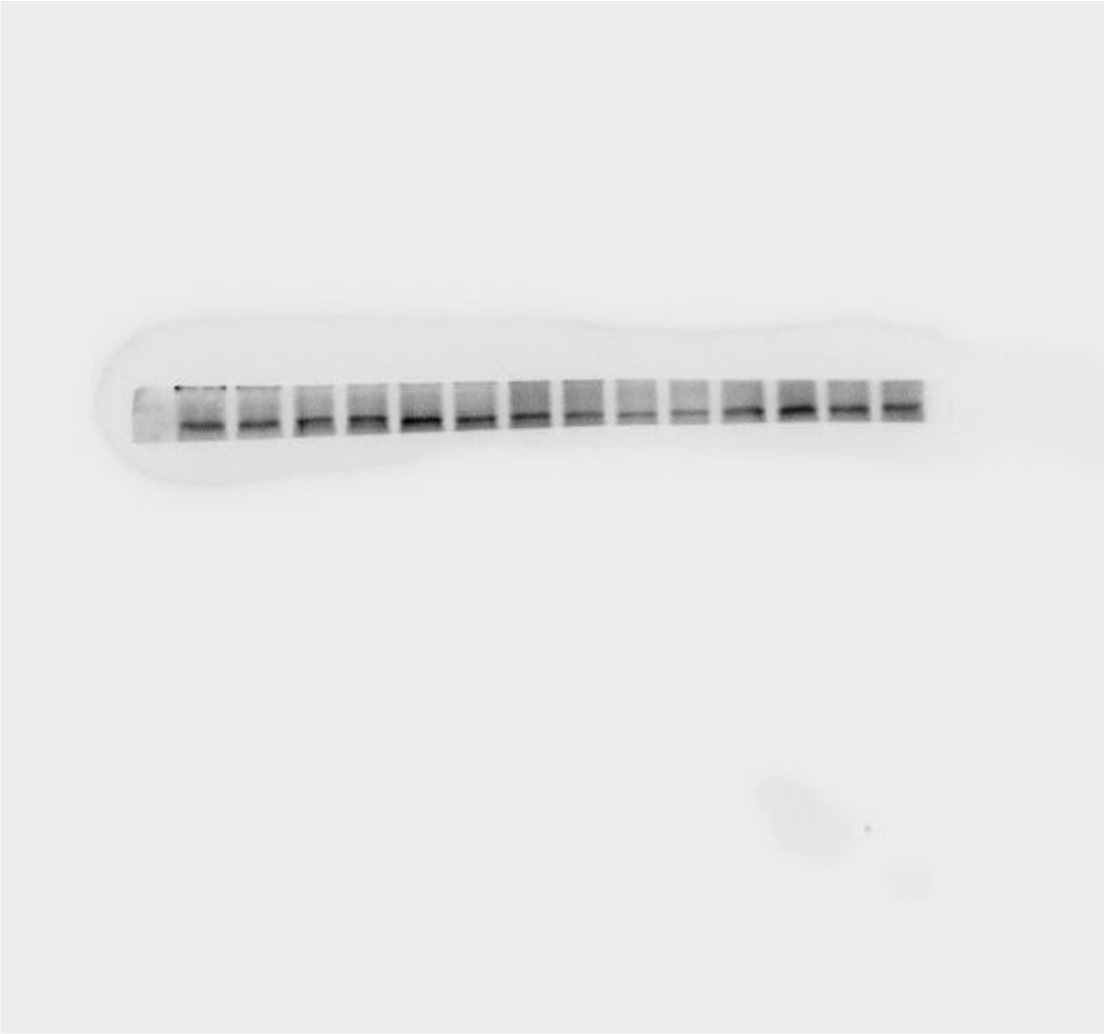

pJNK

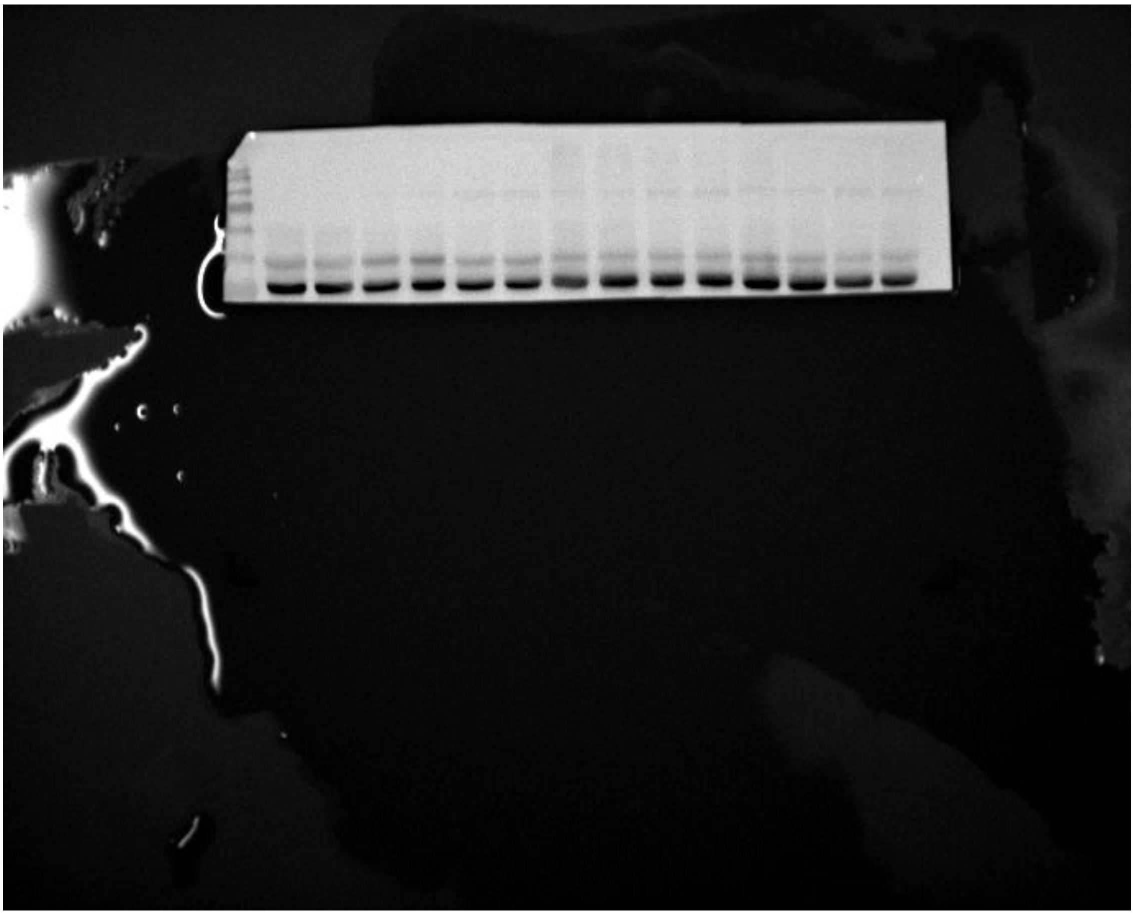

pP38

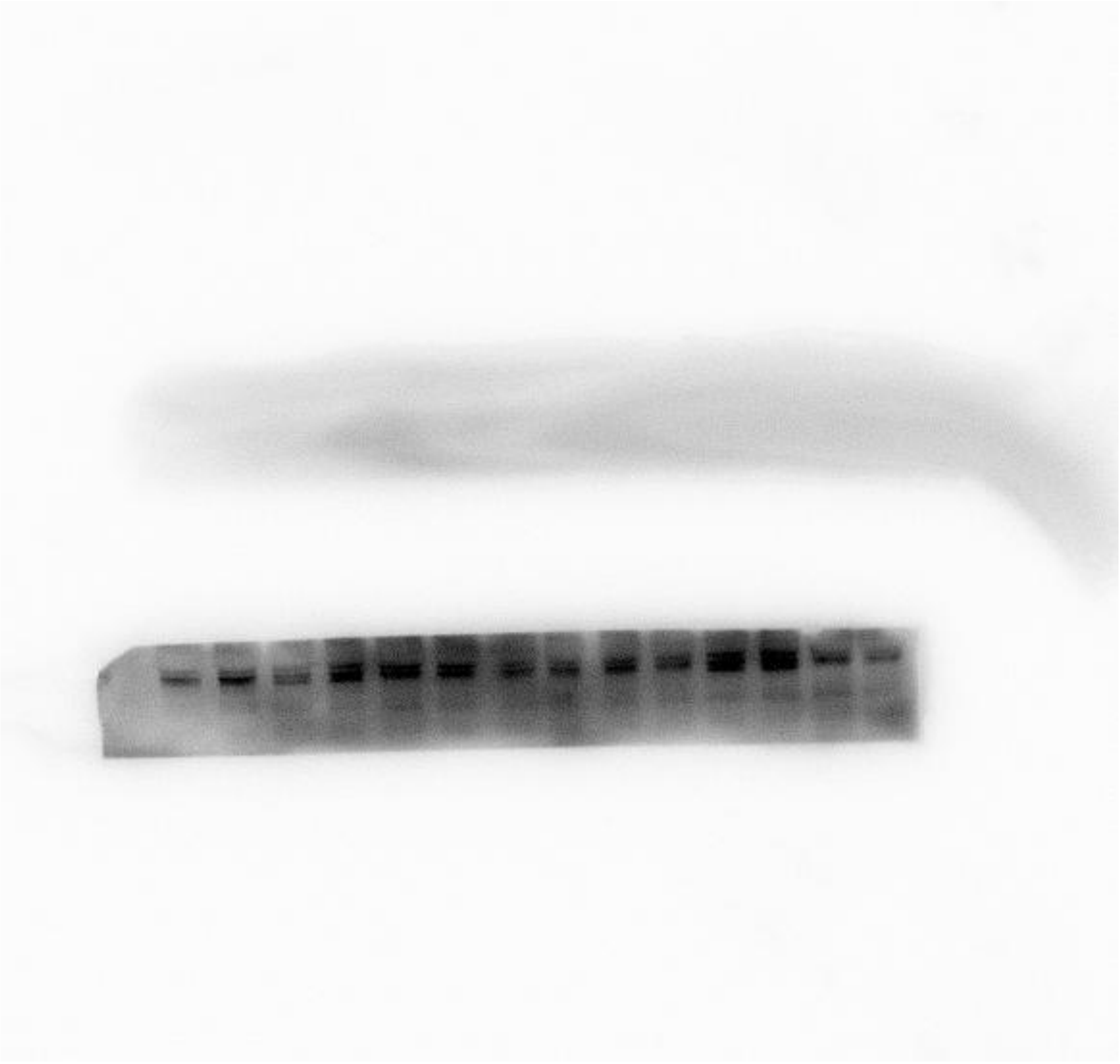

β-actin

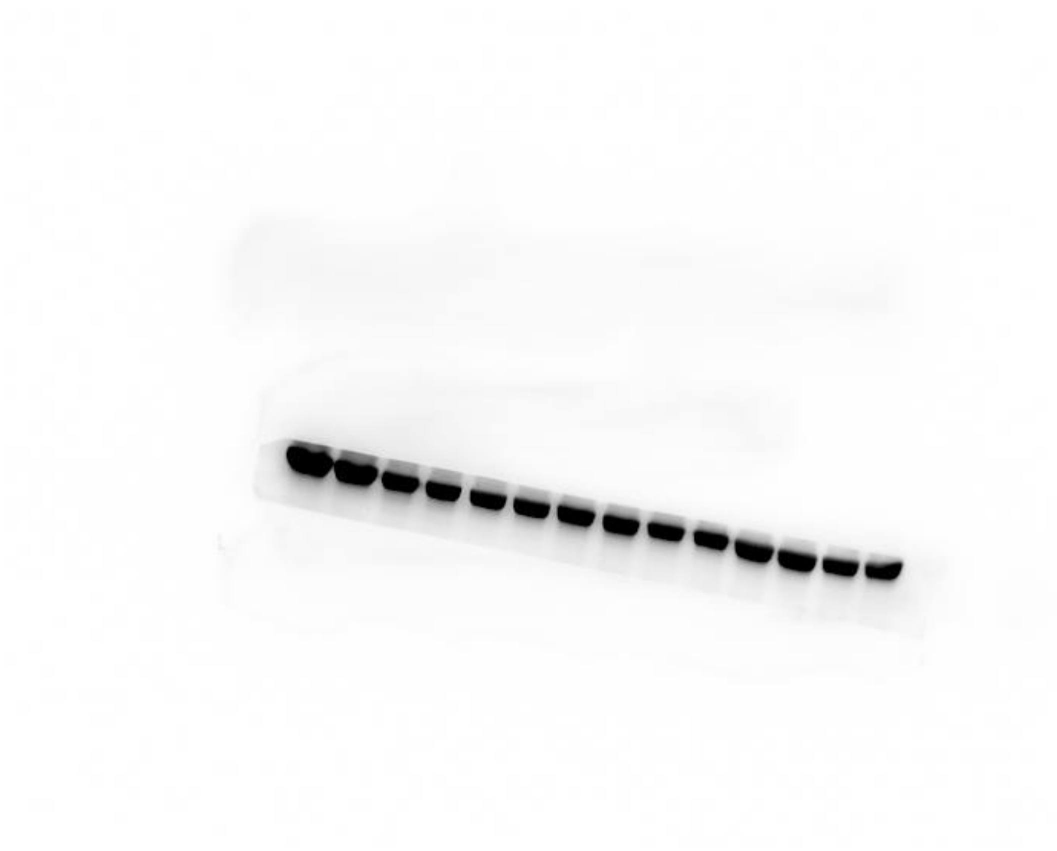

Bip

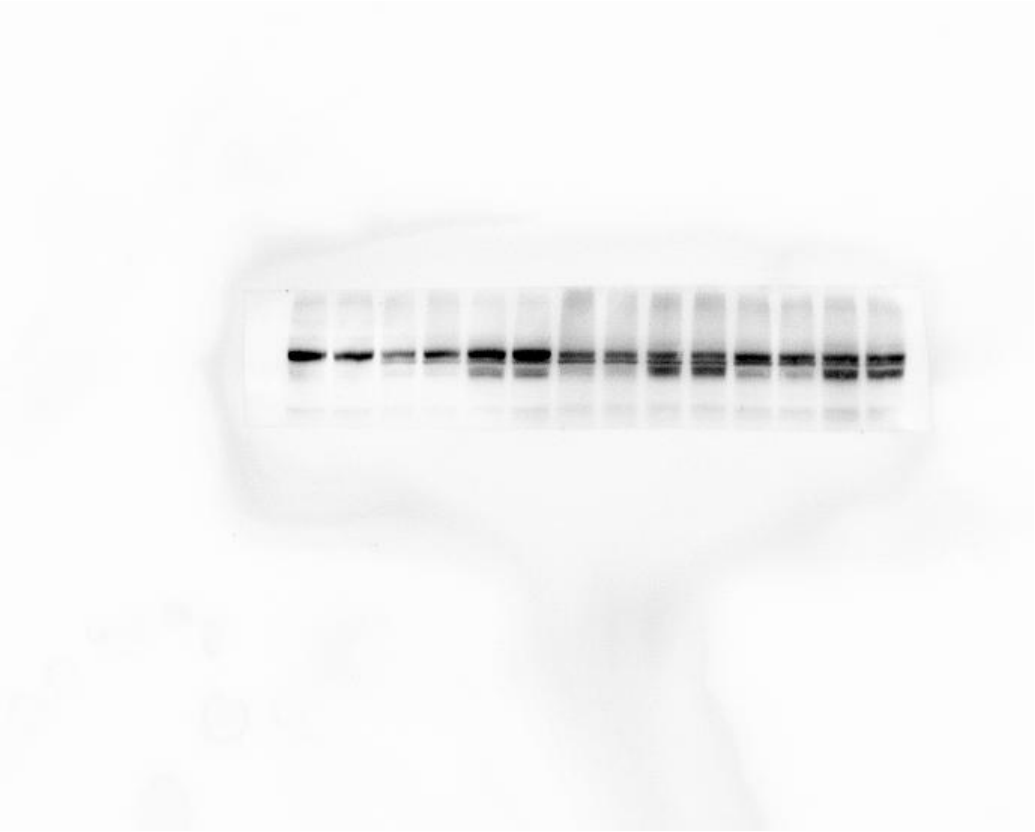

Calnexin

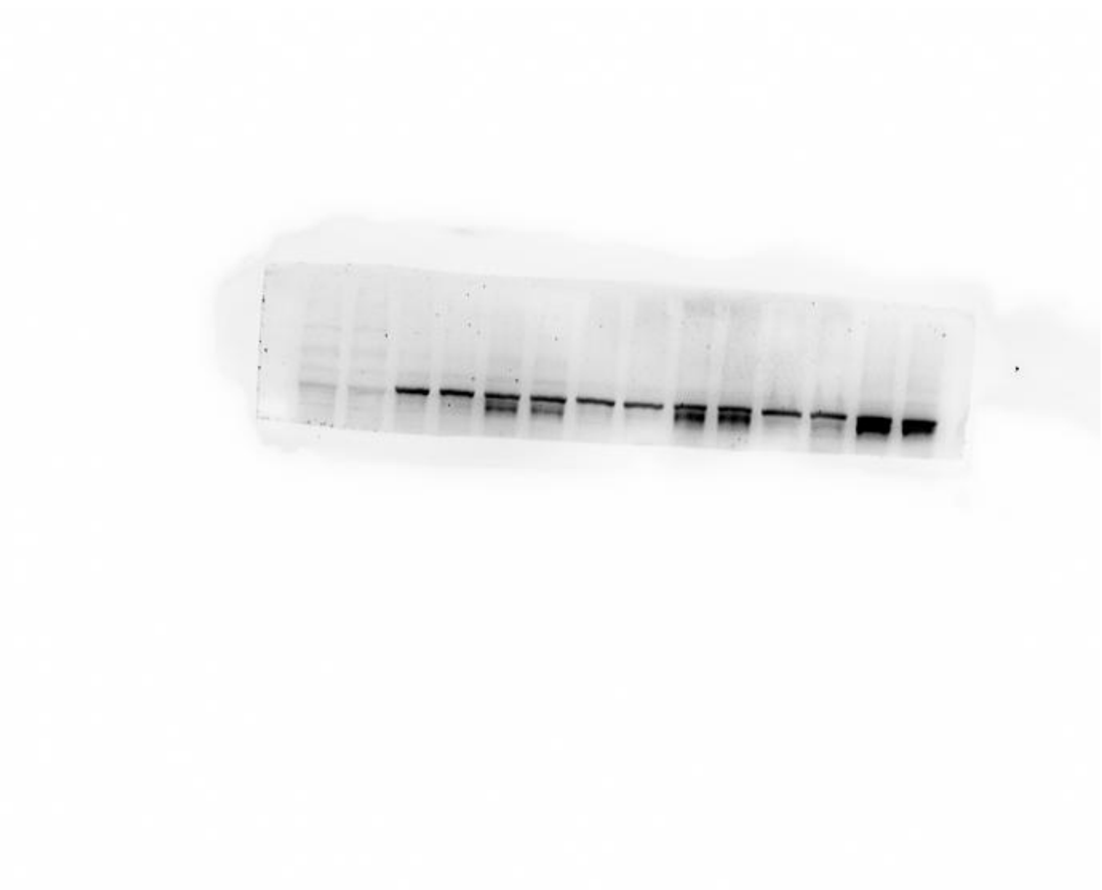

Ero1a

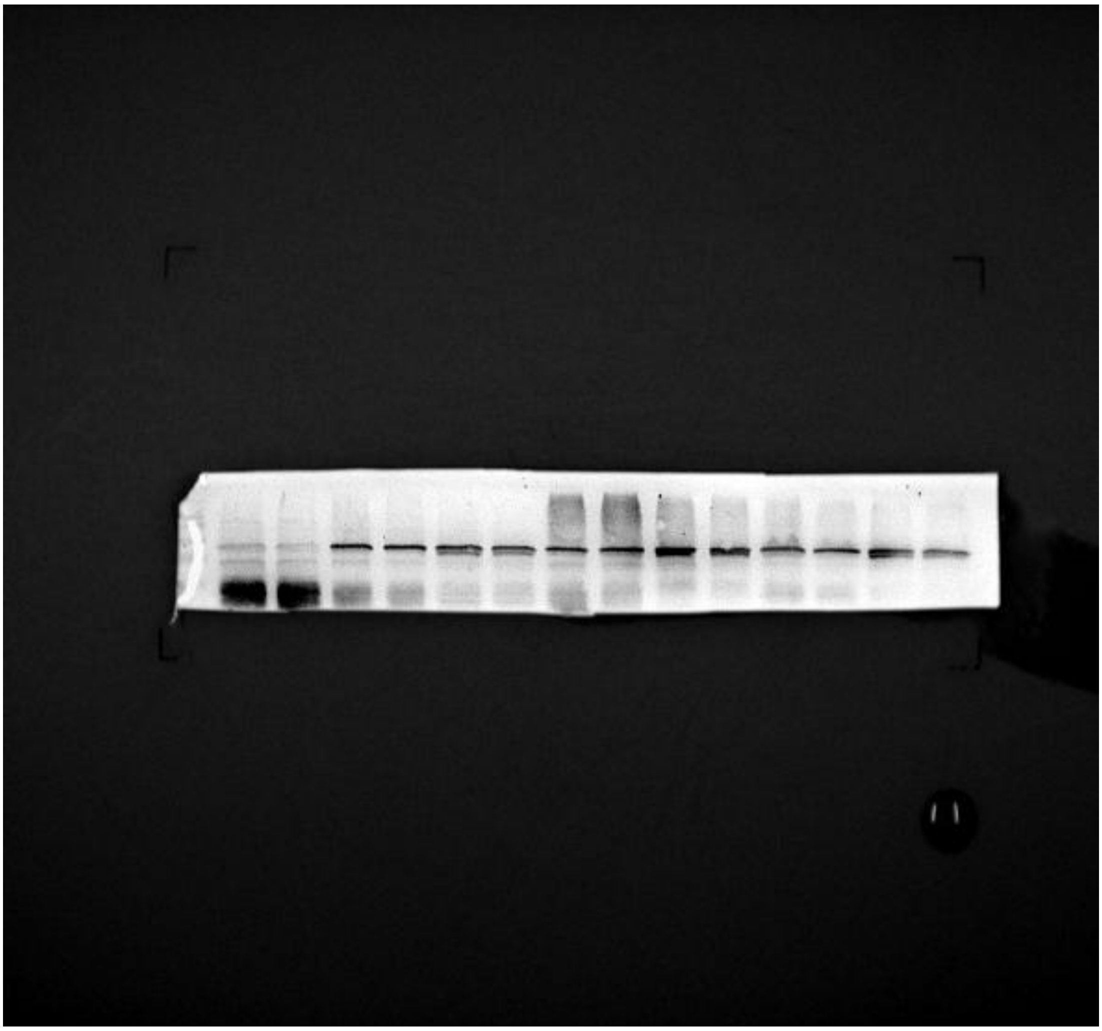

Nrf-2（upper） and β-actin （bottom）

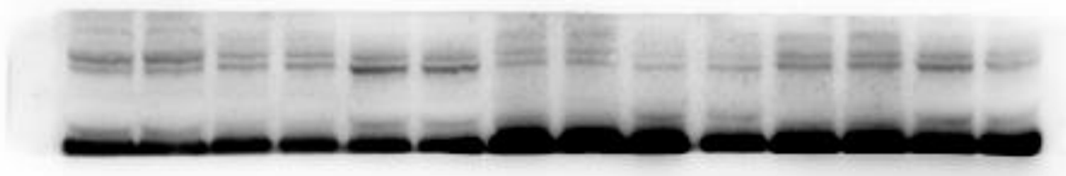

ATF-6

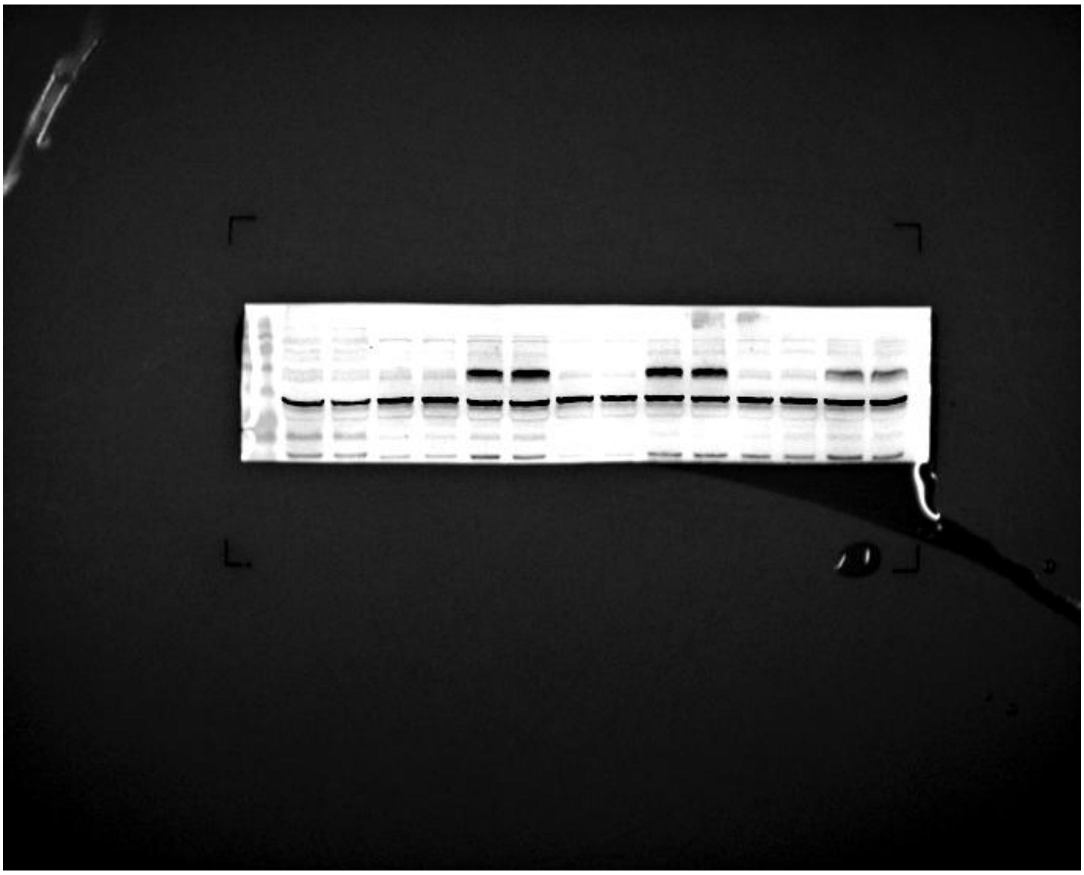

β-actin

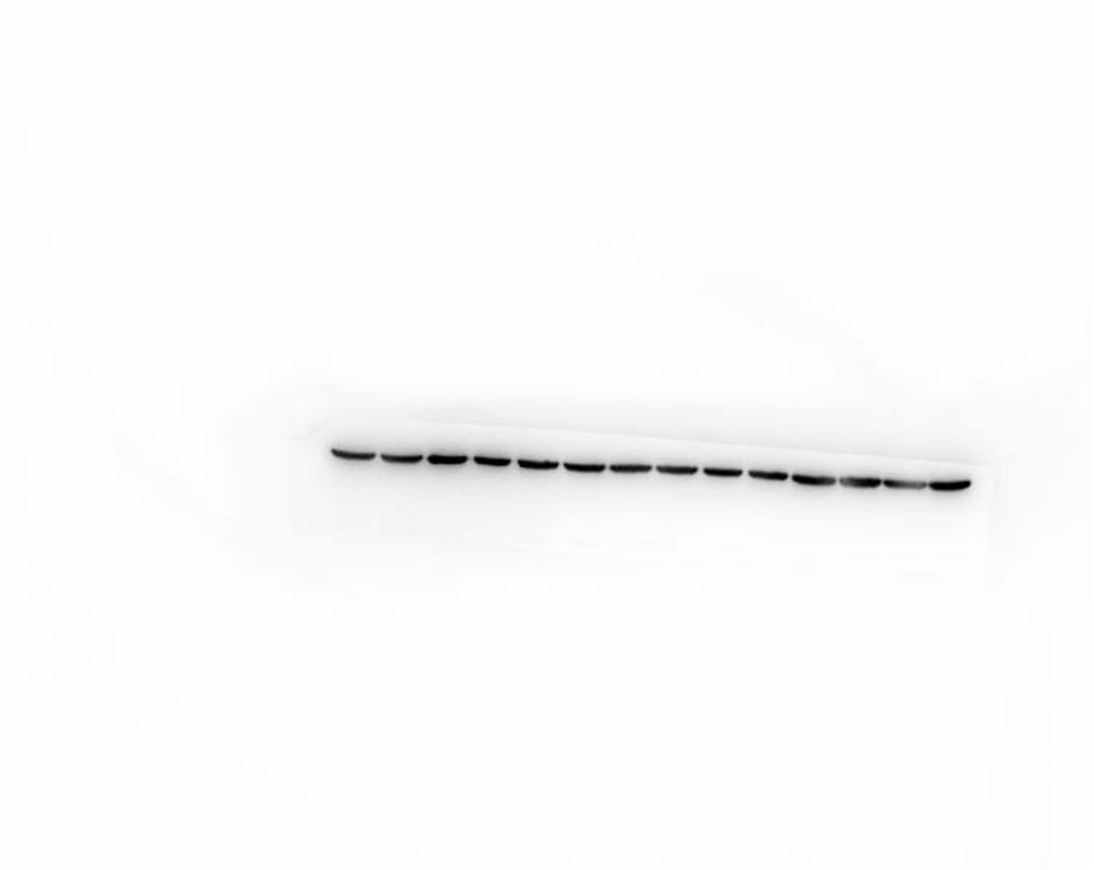

Cleaved-caspase 3

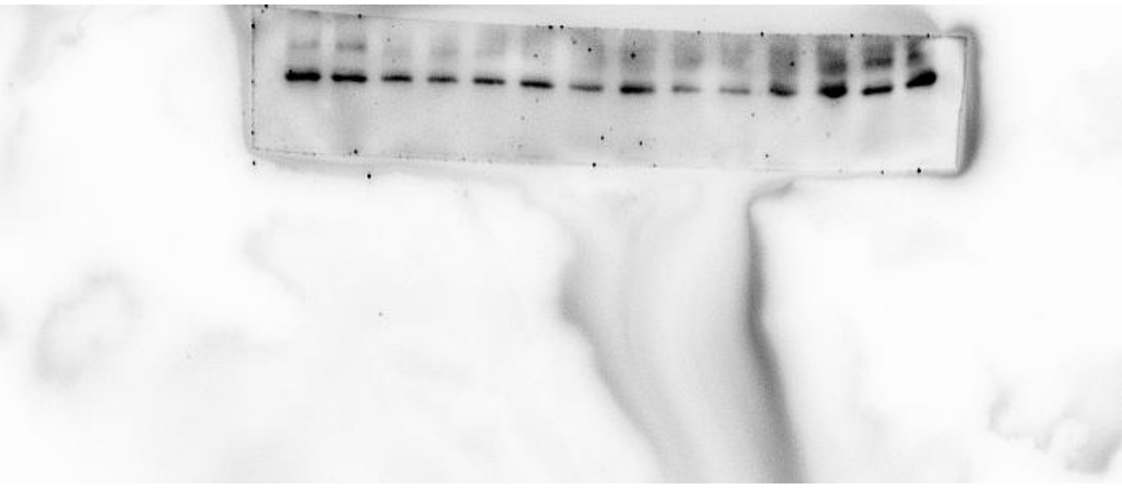

Bcl-2

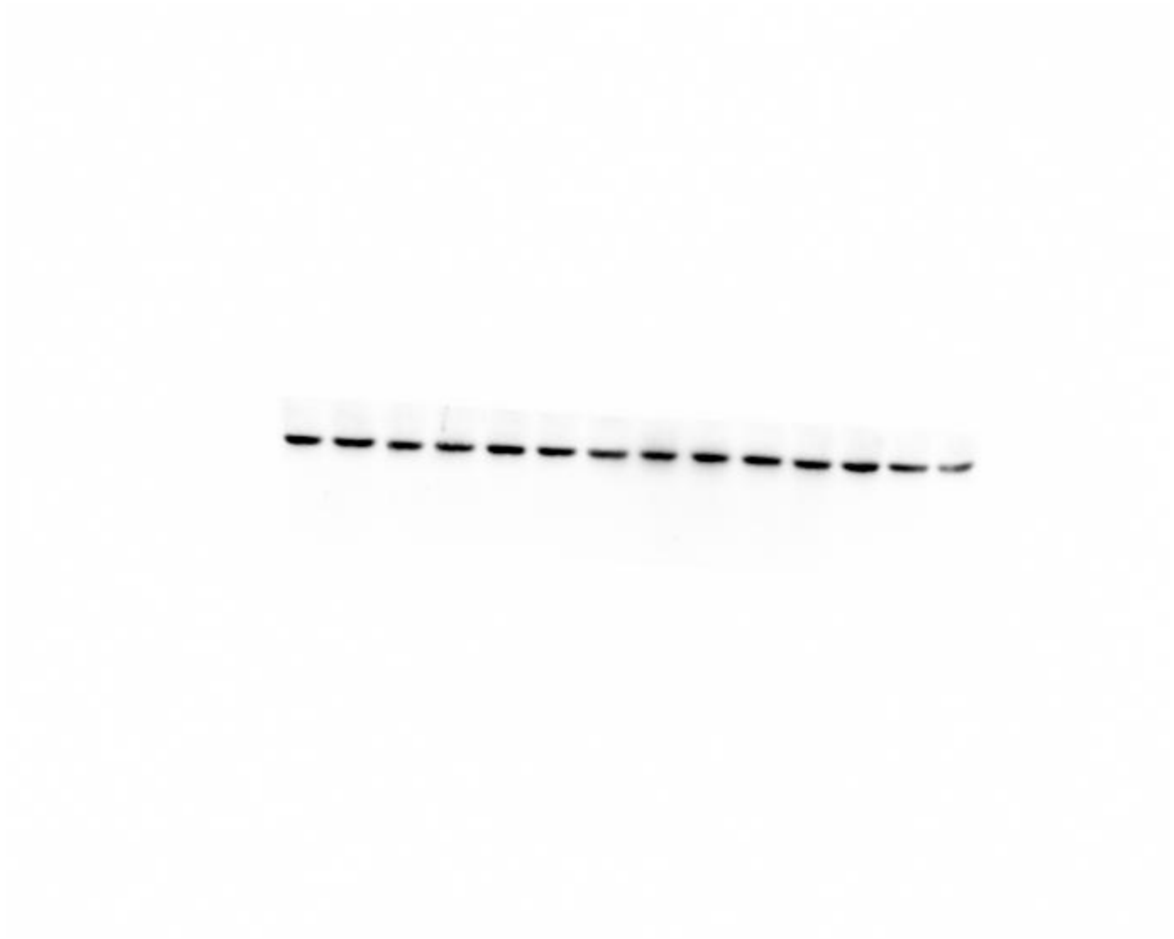

β-actin

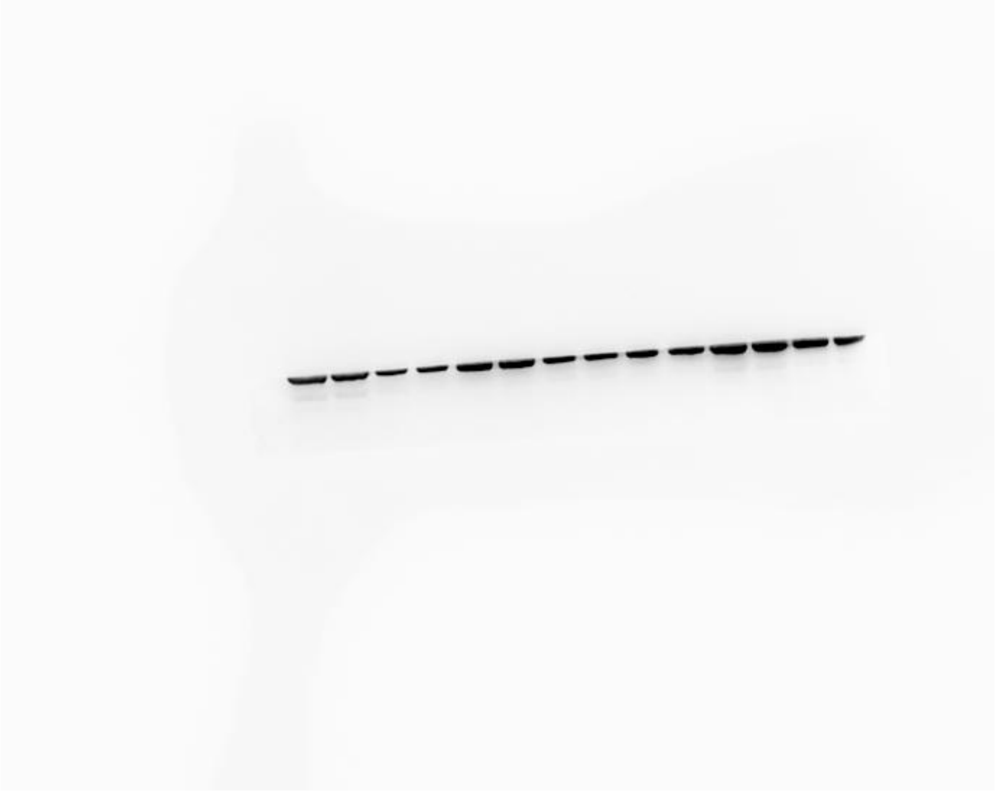

Supplement: DATA SHEET S1 — The whole raw bands of Western Blot exposed using Tanon 2500 Gel Imaging System, including the bands of CD34, VE-cadherin in Figure 2, pmTOR, LC3B, P62, and HIF1α in Figure 5, ER stress pathway protein in Figure 6, and cleaved-caspase 3 and Bcl-2 in Figure 7. [file Data_Sheet_1.pdf]
